# Potential Role of *Domains Rearranged Methyltransferase7* in Starch and Chlorophyll Metabolism to Regulate Leaf Senescence in Tomato

**DOI:** 10.3389/fpls.2022.836015

**Published:** 2022-02-08

**Authors:** Yu Xin Wen, Jia Yi Wang, Hui Hui Zhu, Guang Hao Han, Ru Nan Huang, Li Huang, Yi Guo Hong, Shao Jian Zheng, Jian Li Yang, Wei Wei Chen

**Affiliations:** ^1^State Key Laboratory of Plant Physiology and Biochemistry, College of Life Sciences, Zhejiang University, Hangzhou, China; ^2^Research Centre for Plant RNA Signaling and Zhejiang Provincial Key Laboratory for Genetic Improvement and Quality Control of Medicinal Plants, College of Life and Environmental Sciences, Hangzhou Normal University, Hangzhou, China; ^3^Laboratory of Cell & Molecular Biology, Institute of Vegetable Science, Zhejiang University, Hangzhou, China

**Keywords:** *SlDRM7*, DNA methylation, leaf chlorosis and senescence, starch excess, chlorophyll metabolism

## Abstract

Deoxyribonucleic acid (DNA) methylation is an important epigenetic mark involved in diverse biological processes. Here, we report the critical function of tomato (*Solanum lycopersicum*) *Domains Rearranged Methyltransferase7* (*SlDRM7*) in plant growth and development, especially in leaf interveinal chlorosis and senescence. Using a hairpin RNA-mediated RNA interference (RNAi), we generated *SlDRM7*-RNAi lines and observed pleiotropic developmental defects including small and interveinal chlorosis leaves. Combined analyses of whole genome bisulfite sequence (WGBS) and RNA-seq revealed that silencing of *SlDRM7* caused alterations in both methylation levels and transcript levels of 289 genes, which are involved in chlorophyll synthesis, photosynthesis, and starch degradation. Furthermore, the photosynthetic capacity decreased in *SlDRM7*-RNAi lines, consistent with the reduced chlorophyll content and repression of genes involved in chlorophyll biosynthesis, photosystem, and photosynthesis. In contrast, starch granules were highly accumulated in chloroplasts of *SlDRM7*-RNAi lines and associated with lowered expression of genes in the starch degradation pathway. In addition, *SlDRM7* was activated by aging- and dark-induced senescence. Collectively, these results demonstrate that *SlDRM7* acts as an epi-regulator to modulate the expression of genes related to starch and chlorophyll metabolism, thereby affecting leaf chlorosis and senescence in tomatoes.

## Introduction

Leaf senescence, the final stage of leaf development prior to its death, is a genetically programmed degenerative process, which is accompanied by massive macromolecular catabolism and nutrient recycling to young or storage tissues (Gan and Amasino, 1997; Guo and Gan, 2005). Characterized by leaf chlorosis due to chlorophyll loss, leaf senescence mainly results from age-dependent internal factors, and it also can be triggered by a range of other internal and external cues, including reproduction, phytohormone levels, nutritional signals, water status, light regimes, temperature change, mechanical damage and pathogen attack (Lim et al., 2007a). Although these senescence-influencing factors induce apparently similar phenotypes, the initiation and subsequent processes of senescence are controlled by different molecular modes at multiple regulatory levels (Schippers, 2015; Luo et al., 2018). At the genetic transcriptional and post-transcriptional level, well-established senescence markers include chlorophyll content (Rossi et al., 2015), photochemical efficiency (Guo and Gan, 2005), starch metabolism (Caspar et al., 1985; Zeeman et al., 1998; Zeeman and Rees, 1999), and expression of senescence-associated genes (SAGs) (Lim et al., 2007a). Various transcription factors, such as those belonging to NAC, WRKY, and MYB families (Robatzek and Somssich, 2002; Miao et al., 2004; Ay et al., 2009; Balazadeh et al., 2010; Yang et al., 2014; Lira et al., 2017; Ma et al., 2018; Ma X. et al., 2019; Woo et al., 2019; Jin et al., 2020), have been identified to modulate leaf senescence by activating the expression of downstream SAGs including chlorophyll catabolic genes (CCGs) for chlorophyll degradation (Balazadeh et al., 2010; Woo et al., 2019). In addition, histone modification and chromatin remodeling have been found to regulate certain SAGs expression at the transcriptional level (Lim et al., 2007b; Ay et al., 2009; Chen et al., 2016; Liu et al., 2019), implying a critical role of epigenetic control over leaf senescence.

The main form of conserved epigenetic modification, DNA methylation, often occurs at the 5’ carbon of cytosine base (*^m^*C) and plays roles in genome stability and gene expression (Finnegan and Kovac, 2000; Robertson, 2005). In plant, DNA methylation involves an RNA-directed DNA methylation (RdDM) pathway of *^m^*C establishment in CG, CHG, and CHH (where H is A, C, or T) contexts and *^m^*C maintenance (Zhang et al., 2006; Henderson and Jacobsen, 2007; Law and Jacobsen, 2010). The dynamics of DNA methylation are regulated by DNA methyltransferases and DNA demethylases (Finnegan et al., 1996; Penterman et al., 2007; Lei et al., 2015; Zhang et al., 2018; Liu and Lang, 2020). Plants encode DOMAINS REARRANGED METHYLTRANSFERASE (DRM), METHYLTRANSFERASE (MET), and CHROMOMETHYLASE (CMT) to establish and maintain *^m^*C by distinct pathways (Finnegan and Dennis, 1993; Henikoff and Comai, 1998; Cao and Jacobsen, 2002a). In Arabidopsis (*Arabidopsis thaliana*), MET1 prefers symmetric CG sites, while CMT2 chooses asymmetric CHH sites; CMT3 contributes to maintaining *^m^*CHG and, to a lesser extent, *^m^*CHH; DRM2 establishes *de novo* RdDM and also participates in maintaining *^m^*CHH (Chan et al., 2005; Matzke and Mosher, 2014; Zhang et al., 2018), respectively. Despite the dynamic alternations of global *^m^*C during vegetative and reproductive growth, DNA methylation plays an important role in regulating plant development, reproduction, and responses to biotic and abiotic stresses (Ronemus et al., 1996; Finnegan et al., 2000; Chinnusamy and Zhu, 2009; Slotkin et al., 2009; Dowen et al., 2012; Ay et al., 2014). Defects in RdDM and *^m^*C maintenance showed many phenotypic and developmental abnormalities, including reduced apical dominance, smaller plant size, altered leaf size with curly shape, decreased fertility, and varied flowering time (Finnegan et al., 1996; Kankel et al., 2003; Yang et al., 2019). Although neither the *cmt3* mutants (Lindroth et al., 2001) nor the *drm1 drm2* double mutants (Cao and Jacobsen, 2002b) show morphological differences from wild type (WT), *drm1 drm2 cmt3* plants showed pleiotropic phenotypes including developmental retardation, reduced plant size, and partial sterility (Cao and Jacobsen, 2002a), whereas targeted disruption of rice OsDRM2, which caused a 13.9% decrease in genome-wide *^m^*C, displayed at vegetative and reproductive development in *Oryza sativa*, showing growth defects, semi-dwarfed stature, reductions in tiller number, delayed or no heading, aberrant panicle and spikelet morphology, and complete sterility (Moritoh et al., 2012).

However, limited progress has been made toward elucidating the involvement of DNA methylation during plant aging and senescence. For instance, the transcript levels of *MET1*, *CMT3*, *DRM1*, and *DRM2* are shown to be repressed during leaf senescence (Cao and Jacobsen, 2002a; Jackson et al., 2002; Kankel et al., 2003; Law and Jacobsen, 2010). Additionally, the expression of 16 methylation-associated genes, including *MET1*, *REPRESSOR OF SILENCING 1* (*ROS1*), and *ARGONAUTE 10* (*AGO10*) were significantly downregulated in aging Arabidopsis leaves (Ay et al., 2014). Recently, Arabidopsis *dml3* (DEMETER-Like DNA demethylase3) knockout (KO) mutant results in genome-wide hypermethylation, especially in the promoters of many SAGs whose expression are consequently suppressed, leading to a significant delay in leaf senescence (Yuan et al., 2020). In fact, global *^m^*C decreased dynamically during shoot aging (Ogneva et al., 2016). However, the precise relevance of such epi-modification in controlling leaf senescence, and the pertinent underlying mechanisms are still largely unknown. Here, we report that *SlDRM7* (*Solyc04g005250.2*) impacts chloroplast development *via* modulating starch accumulation and senescence-related chlorophyll synthesis and imposes epi-effects on leaf senescence that affects vegetative growth in tomatoes.

## Materials and Methods

### Plant Materials and Growth Conditions

Wild-type tomato *Solanum lycopersicum* cultivar Ailsa Craig (AC) and the *SlDRM7*-RNAi lines (AC background) were generated and used in this study. Tomato seeds were either germinated directly in compost (Sunshine Mix 3, Sungro Horticulture Canada) or surface-sterilized and germinated on 1/2 Murashige and Skoog (MS) medium for 6 days before being transferred to 1/5 Hoagland solution (pH 5.5) for hydroponic growth. Seedlings that are 5-week-old were then transferred to composts and grown in insect-free growth rooms or greenhouses at 25°C under a 16-h-light/8-h-dark cycle with a humidity of 60 to 80% (Chen et al., 2018a).

### RNA Interference Constructs, CRISPR/CAS9 Gene Editing, and Tomato Transformation

The *SlDRM7* RNAi vector pRNAi-SlDRM7 was constructed as described (Chen et al., 2018b; Yao et al., 2020). A 230-bp *SlDRM7* fragment was PCR-amplified using tomato cDNA as a template and cloned in the sense and antisense orientations into the pRNAi-LIC vector (Chen et al., 2018b). A pair of 20-bp sgRNA oligos targeting the exon of *SlDRM7* was cloned into the plant CRISPR/Cas9-induced vector to produce the SlDRM7 gene editing constructs. Tomato transformation was performed as previously described (Yao et al., 2020). Briefly, the construct was transformed into tomato cotyledons by *Agrobacterium tumefaciens* strain GV3101 to induce shoots under the selection of kanamycin resistance (Dobrev and Kaminek, 2002). Regenerated shoots with 3 to 4 cm length were cut off from independent calli and transferred to the rooting medium for root development (Kit et al., 2010). To confirm the stable transformation event, putatively transformed plantlets with well-developed roots were subjected to molecular analyses through genomic PCR and RT-qPCR. Primers used for making these constructs are listed in Supplementary Table 1.

### Statistical Analysis of Morphological Features

To differentiate WT and *SlDRM7*-RNAi tomato plants, at least 10 leaflets of each line were scanned for measurement of leaf area using ImageJ software. All images showing phenotypes were captured with a Canon digital camera.

### Photosynthetic Pigment Quantification and Confocal Microscopy of Chlorophyll Auto-Fluorescence

About 0.02 g fresh leaf samples collected from the 2nd leaf of six-leaf-stage tomato seedlings were immersed in 10 ml 80% (v/v) acetone in the dark for 24 h until leaves were completely bleached. The absorbance of the supernatant was measured, respectively, at 645, 663, and 470 nm, then chlorophyll a/b and carotenoids content were calculated. The photosynthetic pigment content was calculated as described (Arnon, 1949). Three biological replicates and three technical replicates for each leaf sample were measured. To determine chlorophyll auto-fluorescence, the 2nd leaf of six-leaf-stage tomato seedlings was examined with LSM710nlo laser scanning confocal microscope (Zeiss, Germany).

### Photosynthetic Measurements

The Li-6400 portable photosynthesis system (LI-COR, Lincoln, NE, United States) was used to measure the photosynthetic physiological indexes of the 2nd leaf of six-leaf-stage tomato seedlings. The reference CO_2_ concentration was held at 480 μmol mol^–1^ and leaf temperature at 25°C for all measurements. Air humidity inside the leaf chamber was equivalent to values measured inside the greenhouse (approx. 75%). The light and CO_2_ response curves were measured by varying light intensity from 0 to 2,500 μmol ⋅ m^–2^ ⋅ s^–1^. Net photosynthesis rate (Pn), respiration rate, transpiration rate (Tr), stomatal conductance (Gs), and intercellular CO_2_ concentration (Ci) were measured at 2,000 μmol ⋅ m^–2^ ⋅ s^–1^. Five replicates were measured at each light intensity. The light and CO_2_ response curves were simulated by a non-rectangular hyperbola model:


$$\text{An}\left(I\right)=\frac{\alpha {\text{IA}}_{\max}-\sqrt{{\left(\alpha {\text{IA}}_{\max}\right)}^{2}-4\theta \alpha {\text{IA}}_{\max}}}{2\theta }-{\text{R}}_{d}$$

An(I): Pn, I: light intensity, θ: the initial slope, α: the initial photochemical efficiency, Rd: dark respiration (Thornley, 1976).

### Transmission Electron Microscopy

About 1 × 3 mm^2^ leaf tissues were cut off from the seedlings at the six-leaf stage. Natural senescence leaf samples were collected from WT. Leaf samples were first fixed with 2.5% glutaraldehyde in phosphate buffer (0.1 M, pH 7) for more than 4 h, washed three times in the phosphate buffer for 15 min each, then post-fixed with 1% OsO_4_ in phosphate buffer for 1–2 h, and washed three times in the phosphate buffer. Leaf samples were first dehydrated by a graded series of ethanol and then dehydrated by pure acetone. Next, the specimen was placed in a 1:1 mixture of absolute acetone and the final *Spurr* resin mixture for 1 h at room temperature, then transferred to a 1:3 mixture of absolute acetone, and the final resin mixture for 3 h and transferred to final *Spurr* resin mixture for overnight. The specimen was placed in Eppendorf which contained *Spurr* resin and heated at 70°C for 9 h. The specimen was sectioned in LEICA EM UC7 ultratome and sections were stained by uranyl acetate and alkaline lead citrate for 5 to 10 min, respectively, and observed under a Hitachi Model H-7650 TEM (Hitachi, Japan).

### Histological Detection of Starch and Measurements of Starch Content

Leaves were treated with 80% (v/v) ethanol to remove chlorophylls and stained with Lugol’s iodine solution to detect starch distribution (Tsai et al., 2009). Images were captured with a Canon digital camera.

Starch content was measured following the method as described by Clegg (1956). Briefly, 0.02 g of fresh leaves were ground in liquid nitrogen. Leaf powders were mixed with 80% ethanol and then incubated at 60°C for 20 min. After centrifugation at 4,000 rpm/min for 5 min, the supernatant was discarded. The samples were then resuspended in 3 ml of ddH_2_O and 2 ml of 9.2 M perchloric acid and incubated at 100°C for 10 min followed by centrifugation at 4,000 rpm/min for 10 min. This step was repeated three times and the supernatant was pooled. The 5 ml of anthrone reagent was added to a 0.1 ml aliquot of extract for glucose measurement. The intensity of the color formed was measured at 620 nm after heating on a boiling water bath for 10 min and rapidly cooled. The glucose concentration was estimated using a standard curve prepared from different glucose concentrations. Since 0.9 g starch yields approximately 1 g of glucose on hydrolysis, the conversion factor is 0.9 for the starch extract.

### Total RNA Extraction and Quantitative RT-PCR Analyses

Total RNA was extracted from the 2nd leaf of six-leaf-stage tomato seedlings using the RNAprep pure Plant Kit (Tiangen). Then, quantitative real-time PCR (RT-qPCR) was carried out on a LightCycler480 machine (Roche Diagnostics, Switzerland) using SYBR Premix Go Taq by CFX96™ Real-Time System (Bio-Rad, United States). The relative expression level of genes was calculated using the formula 2^–ΔΔCt^ and normalized to the amount of *Actin* mRNA detected in the same samples. At least three technical replicates for each of three biological replicates for each sample were performed in this study. All primers used for real-time PCR analysis were listed in Supplementary Table 1.

### Deoxyribonucleic Acid Extraction and Whole-Genome Bisulfite Sequencing

Genomic DNA was isolated from the 2nd leaf of six-leaf-stage tomato seedlings harvested from WT or *SlDRM7*-RNAi lines using the DNeasy Plant Mini Kit (Qiagen). Two biological replicates for each sample were performed. About 100 ng genomic DNA spiked with 0.5 ng lambda DNA were fragmented by sonication to 200–300 bp with Covaris S220. These DNA fragments were treated with bisulfite using EZ DNA Methylation-Gold™ Kit (Zymo Research). The library was constructed by Novogene Corporation (Beijing, China), and sequenced on the Illumina Novaseq platform (United States). Image analysis and base calling were performed with Illumina CASAVA pipeline and finally generated 150-bp paired-end reads. The FastQC (fastqc_v0.11.5) was used to perform basic statistics on the quality of the raw reads, which were pre-processed through fastp (fastp 0.20.0). The remaining reads that passed all the filtering steps, counted as clean reads, were mapped to the reference tomato genome^1^ by BSMAP. The tomato genome fasta were obtained from Ensemble Plants^2^. The reference genome and clean reads were transformed into bisulfite-converted version (C-to-T and G-to-A converted) and then indexed using bowtie2 (Langmead and Salzberg, 2012). Sequence reads that produce a unique best alignment were then compared to the normal genomic sequence and the methylation state of all cytosine positions was inferred. The sequencing depth and coverage were summarized using deduplicated reads.

### Methylation Analysis

Results of methylation extractor were transformed into bigWig format for visualization using IGV browser. The sodium bisulfite non-conversion rate was calculated as the percentage of cytosine sequenced at cytosine reference positions in the lambda genome. Methylated sites were identified with a binomial test using the methylated counts (mC), total counts (mC+unmC), and the non-conversion rate (r). Sites with FDR-corrected *P* value < 0.05 were considered methylated sites. To calculate the methylation level of the sequence, Methylation Level (ML) is defined as ML(C) = *reads*(*mC*)/[reads(mC)reads(C)].

Differentially methylated regions (DMRs) were identified using the DSS, which is a new dispersion shrinkage method for estimating the dispersion parameter. According to the distribution of DMRs through the genome, we defined the genes related to DMRs as genes whose gene body region (from TSS to TES) or promoter region (2 kb upstream from the TSS) have an overlap with the DMRs. *P*-values less than 0.05 were considered significantly enriched by DMR-related genes.

### RNA-Seq and Data Analysis

Total RNA was isolated from the same leaf materials as for the DNA extraction. Three biological replicates for each sample were performed. Five micrograms of pooled RNA were used for the RNA-seq library using the Illumina Genome Analyzer (Solexa, United States). The sequencing data was filtered with SOAPnuke (v1.5.2). Low-quality reads were removed from the raw data, and high-quality reads were aligned to the tomato genome (version SL2.50; see text footnote 1). Differential expression analysis was performed using the DESeq2 (Love et al., 2014) with a corrected *P*-value (*q* value) < 0.05. To take insight to the change of phenotype, GO^3^ and KEGG^4^ enrichment analysis of annotated different expressed genes was performed by Phyper^5^ based on Hypergeometric test. The significant levels of terms and pathways were corrected by *q* value with a rigorous threshold (*q* value < 0.05) by Bonferroni.

### Statistical Analysis

All data in this study were presented as mean ± standard deviation (SD). Student’s *t*-test or One-Way ANOVA followed by multiple comparison (Tukey’s HSD, *P* ≤ 0.05) was performed to analyze the significant difference between genotypes.

## Results

### *SlDRM7* Is Essential for Tomato Leaf Development and Vegetative Growth

**FIGURE 1:**
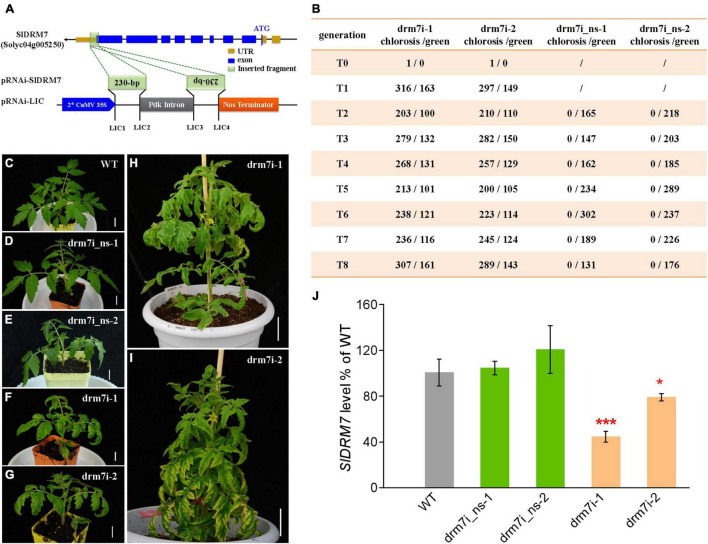
Generation and characterization of tomato *SlDRM7*-RNAi lines. **(A)** Schematic of the pRNAi-SlDRM7 construct. A 230-bp fragment of *SlDRM7* (Solyc04g005250.2) was cloned into the pRNAi-LIC vector to generate pRNAi-SlDRM7. **(B)** Summary of segregation of leaf chlorosis/senescence phenotype in T0 to T8 generations of *SlDRM7*-RNAi lines. The numerator represents the number of progenies of *SlDRM7*-RNAi lines with maintained interveinal chlorosis/senescence leaves with kanamycin resistance, while the denominator represents the number of progenies with normal green leaves with kanamycin sensitivity. **(C–I)** Segregation of leaf chlorosis in *SlDRM7*-RNAi lines. wild-type (WT) seedlings display normal green leaves **(C)**, while progenies of *SlDRM7*-RNAi lines display normal WT green leaves called drm7i_ns-1 **(D)**, and drm7i_ns-2 **(E)**, whereas progenies maintain interveinal chlorosis/senescence leaves called drm7i-1 **(F,H)** and drm7i-2 **(G,I)**. Tomato seeds were spread and germinated directly in compost, and seedlings were photographed at 10 days after germination in **(C–G)** with bars = 1 cm, while seedlings were photographed at 6 weeks after germination in **(H,I)** with bars = 5 cm. **(J)** RT-qPCR analysis of the relative expression levels of *SlDRM7* in leaves of WT and *SlDRM7*-RNAi lines at the six-leaf stage. Data are means ± SD of five biological replicates. Asterisks indicate the significant differences compared with WT (*P ≤ 0.05, ***P ≤ 0.001, one-way ANOVA, Tukey’s HSD). No difference with statistical significance was found for WT vs. drm7i_ns-1, and WT vs. drm7i_ns-2.

Through a gene-specific RNAi strategy (Figure 1A), we generated two independent tomato *SlDRM7*-RNAi lines drm7i-1 and drm7i-2. Compared with WT, *SlDRM7*-RNAi resulted in leaf interveinal chlorosis and senescence in T0 and T1 to T8 seedlings of the two independent drm7i lines (Figures 1B–I). The chlorotic phenotype along with antibiotic resistance exhibited Mendelian segregation, displaying approx. 2:1 ratio of chlorotic to normal green leaves among viable seedlings in T1, and continuing to segregate in T2 to T8 generations of both lines, while seedlings without the transgene after segregation in T1 to T8 reversed to green and were sensitive to antibiotic selection (Figure 1B). Intriguingly, no homozygous transgenic plants were obtained for both *SlDRM7*-RNAi lines. These data indicate that the dominant leaf chlorotic phenotype is genetically linked with the presence of a single copy of the pRNAi-SlDRM7 transgene, presumably resulting from the RNAi-mediated specific suppression of *SlDRM7* expression rather than non-specific off-target effect in heterozygous plants of the two *SlDRM7*-RNAi lines. Homozygous *SlDRM7*-RNAi lines may be lethal to survival (due to extreme senescence). Considering the critical role of SlDRM7 in *de novo* RdDM, it is possible that *SlDRM7*-RNAi may impose some epigenetic remodeling of its target genes that are required for proper leaf development, and such epigenetic remodeling to control leaf chlorosis is not *trans-*generationally heritable but relies on constant RNAi of *SlDRM7*.

To investigate the genetic bridge between the genotype and phenotype in drm7i lines, we used drm7i_ns-1 and drm7i_ns-2 that were reverted to WT non-segregating (ns) green leaves after segregation as negative controls. Compared to WT and drm7i_ns lines, endogenous SlDMR7 expression level was dramatically reduced by approximately 30–60% in drm7i lines (Figure 1J). When the *SlDRM7*-RNAi affected vegetative leaf development, slight chlorosis started in the margin of the newly formed leaves of drm7i-1 during the 1st week transferred into a hydroponic culture, became more pronounced by the end of 2nd week, and gradually spread toward the interveinal regions, resulting in the characteristic phenotype of leaf interveinal chlorosis (Supplementary Figure 1A). We also generated *SlDRM7*-knockout (KO) lines where CRISPR/Cas9-induced gene editing resulted in nucleotide deletions of SlDRM7 (Supplementary Figure 1B). To our surprise, no defect in compound leaf development with chlorotic phenotype was observed in both KO lines vs. drm7i-1 (Supplementary Figures 1C–H). Such striking phenotypic differences between *SlDRM7*-RNAi and *SlDRM7*-KO lines are not unprecedented with genes essential for development and growth and are often associated with “transcriptional adaptation” or “genetic compensation response” (El-Brolosy et al., 2019; Ma Z.P. et al., 2019; Wang et al., 2020), which may be able to offset the effects of a completely dysfunctional *SlDRM7* gene that has lost its capacity to express SlDRM7 protein in KO lines. To test this possibility, we analyzed the expression of *SlDRM5*, *SlDRM6*, and *SlDRM8* in KO lines. Compared to *SlDMR6* and *SlDMR8*, *SlDRM5* was almost not expressed in tomato leaf. While the expression of *SlDRM8* was not affected by the knockout of SlDRM7, that of *SlDMR6* was significantly induced (Supplementary Figure 2).

In addition, *SlDRM7*-RNAi also affected the leaf size and plant growth at the vegetative stage (Supplementary Figure 3). At the six-leaf stage, we measured the leaf area of the first three compound leaves and found that the average lobule area of leaves was predominant in WT, followed by that in drm7i_ns lines, while the smallest in drm7i lines (Supplementary Figures 3A–C). When checking the shoot height of seedlings after 4-week hydroponic culture, we found that the average shoot height was about 16.84 (±2.18) cm in WT, 10.85 (±1.81) cm and 10.10 (±1.44) cm in drm7i_ns-1, and drm7i_ns-2, while only 7.72 (±0.99) cm and 8.63 (±0.99) cm in drm7i-1 and drm7i-2, respectively (Supplementary Figures 3D–F), consistent with the stunted plant growth exhibiting a dwarf and bushy phenotype in drm7i lines (Figures 1H,I). Phenotypically, the distinguishable differences in terms of leaf size and plant height between WT and drm7i_ns, as well as between drm7i_ns and drm7i, suggest that these phenotypic changes may result from *SlDRM7*-RNAi mediated epigenetic modification(s) that can be maintained in the absence of a pRNAi-SlDRM7 trigger.

Taken together, these results demonstrated that the *SlDRM7*-directed mechanism plays an essential role in tomato vegetative development, especially leaf development and senescence. In this work, using *SlDRM7*-RNAi lines drm7i-1 and drm7i-2 and the segregated lines with WT green leaves drm7i_ns-1 and drm7i_ns-2, we focus on understanding how *SlDRM7* governs leaf chlorosis/senescence during vegetative growth.

### *SlDRM7* Modulates Leaf Senescence and the Expression of Senescence-Associated Genes

**FIGURE 2:**
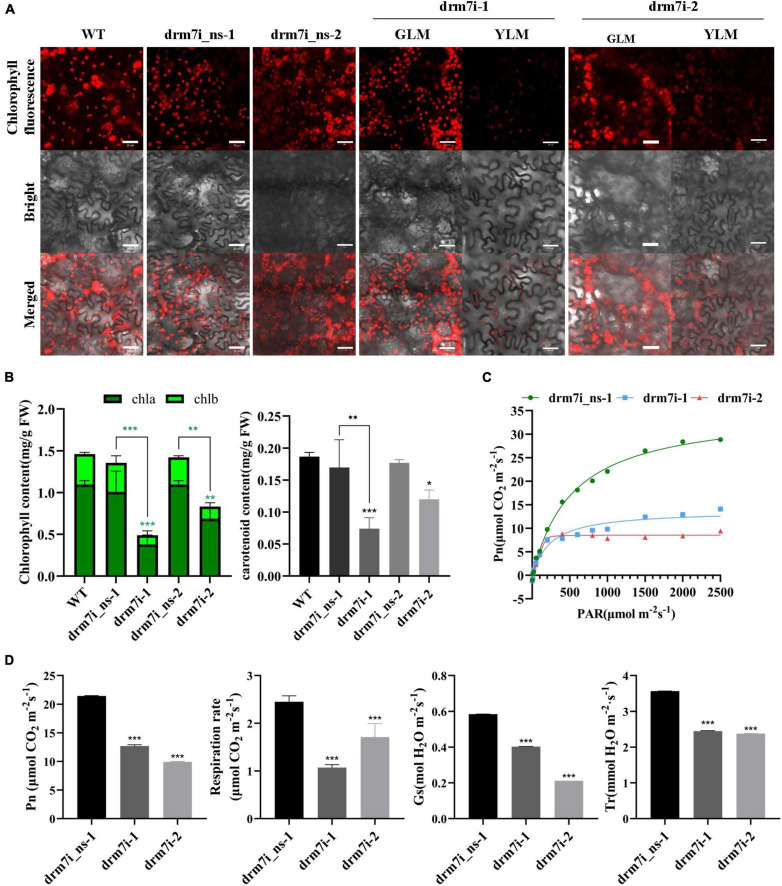
Measurement of photosynthetic pigment content and photosynthetic capacity. **(A)** The auto-fluorescence of chlorophyll in leaves of *SlDRM7*-RNAi lines. Strong chlorophyll auto-fluorescence intensity with almost no differences was observed in leaf Mesophyll cells from the 2nd compound leaves of wild-type AC (WT), two drm7i_ns lines, and the greening leaf Mesophyll (GLM) cells of two drm7i lines at the six-leaf stage, but much weaker in the yellowing leaf mesophyll (YLM) cells of two drm7i lines. WT, drm7i_ns-1 and drm7i_ns-2, drm7i-1 and drm7i-2 are shown. Bars = 20 μm. **(B)** The content of three photosynthetic pigments in leaf tissues of *SlDRM7*-RNAi lines. The content of chlorophyll a (chla) and chlorophyll b (chlb) (left panel), and carotenoid (right panel) was measured in the 2nd compound leaves of six-leaf-stage seedlings of WT, two drm7i_ns lines, and two drm7i lines, respectively. **(C,D)** Effect of *SlDRM7*-RNAi on photosynthesis. Light and CO_2_ response curves (C), as well as net photosynthetic rates (Pn), respiration rate, stomatal conductance (Gs), and transpiration rate (Tr) **(D)** of the 2nd compound leaves of six-leaf-stage seedlings of drm7i_ns-1 and two drm7i lines. Data are means ± SD (*n* = 3 in panel **B**, while *n* = 5 in panel **D**). Asterisks indicate the significant differences when compared with WT or drm7i_ns lines (*P ≤ 0.05, **P ≤ 0.01, ***P ≤ 0.001, one-way ANOVA, Tukey’s HSD).

The leaf chlorotic phenotype prompted us to investigate the function of SlDRM7 in photosynthetic capacity physiologically. At the six-leaf stage, we measured chlorophyll auto-fluorescence, photosynthetic pigment content, and photosynthetic efficiency in the 2nd compound leaves. In yellowing leaf mesophylls (YLM), but not greening leaf mesophylls (GLM) of two drm7i lines, a significant decrease in chlorophyll auto-fluorescence intensity was observed (Figure 2A). Correspondingly, the content of chlorophyll a, chlorophyll b, and carotenoid were significantly lower in these two drm7i lines vs. WT or drm7i_ns lines (Figure 2B). Since there was no obvious difference of chlorophyll auto-fluorescence and photosynthetic pigment content among WT and two drm7i_ns lines (Figures 2A,B), the influence of *SlDRM7*-RNAi on photosynthetic capacity was studied by comparing drm7i lines with drm7i_ns-1. Based on the light and CO_2_ response curves (Figure 2C), the fitted maximum photosynthetic rate (Pn) of drm7i_ns-1 was 35.53 μmol ⋅ m^–2^ ⋅ s^–1^, whereas that of drm7i-1 and drm7i-2 were reduced to only 14.34 and 8.86 μmol ⋅ m^–2^ ⋅ s^–1^, respectively (Figure 2D). Similarly, a decreasing tendency of the respiration rate, stomatal conductance (Gs), and transpiration rate (Tr) were further observed in both drm7i lines (Figure 2D). These data suggest that photosynthesis capacity was inhibited in drm7i vs. drm7i_ns.

**FIGURE 3:**
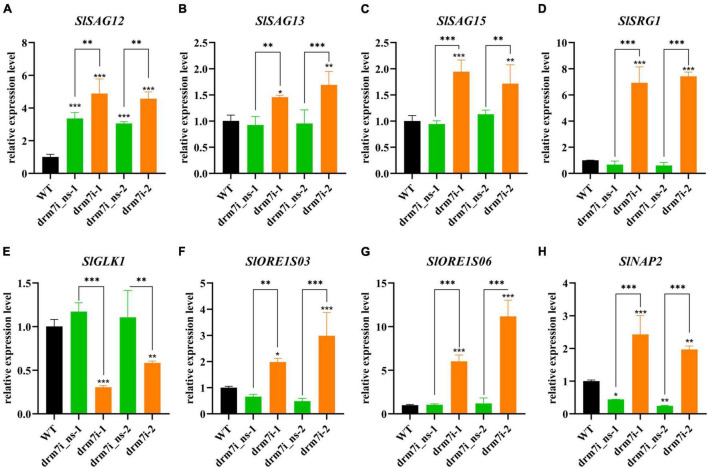
Expression analysis of senescence-associated genes in WT and *SlDRM7*-RNAi leaf tissues. **(A–H)** RT-qPCR analysis of the relative expression levels of *SlSAG12* **(A)**, *SlSAG13* **(B)**, *SlSAG15* **(C)**, *SlSRG1* **(D)**, *SlGLK1* **(E)**, *SlORE1S03* **(F)**, *SlORE1S06* **(G)**, *SlNAP2* **(H)** in mature leaves of the WT, two drm7i_ns lines, and two drm7i lines, respectively. Data are means ± SD of three biological duplicates. Asterisks (above error bar represent the difference with WT; above the short line represent the difference with drm7i_ns lines) indicate the significant differences (*P ≤ 0.05, ^**^P ≤ 0.01, ^***^P ≤ 0.001, one-way ANOVA, Tukey’s HSD).

The decreases in both chlorophyll content and photosynthetic efficiency led us to speculate whether the chlorosis of drm7i lines is associated with premature senescence. Therefore, we examined the expression of SAGs including *SlSAG12*, *SlSAG13*, *SlSAG15*, *SENESCENCE-RELATED GENE1* (*SlSRG1*), senescence-related transcription factors (TFs) genes such as *SlORE1S03*, *SlORE1S06*, and *SlNAP2*, and *GOLDEN2-like* (*SlGLK1*) which is related to chlorophyll biosynthesis (Lira et al., 2017; Ma et al., 2018). Except for *SlGLK1*, the expression of the rest of the genes was upregulated in drm7i lines compared with WT or drm7i_ns lines (Figure 3). Furthermore, we tested the *SlDRM7* expression during age-dependent and dark-induced senescence in WT plants and found that the transcript level of *SlDRM7* was significantly upregulated in senescence leaves induced either naturally or darkly (Supplementary Figure 4). This finding seems contradictory to the genome-wide demethylation during plant senescence (Ogneva et al., 2016), suggesting a putative self-feedback pathway may be involved in regulating senescence by increasing *SlDRM7* expression and a subsequently enhanced epi-control. Taken together, these findings reveal that *SlDRM7* is required for proper vegetative growth, where *SlDRM7* may work as a negative epi-regulator to repress the transcriptional expression of senescence-associated genes, leading to sustain a photosynthetic capacity and consequently inhibit the initiation of leaf senescence in tomatoes.

### Effect of *SlDRM7* on Genome-Wide Methylome and Transcriptional Profiling

**FIGURE 4:**
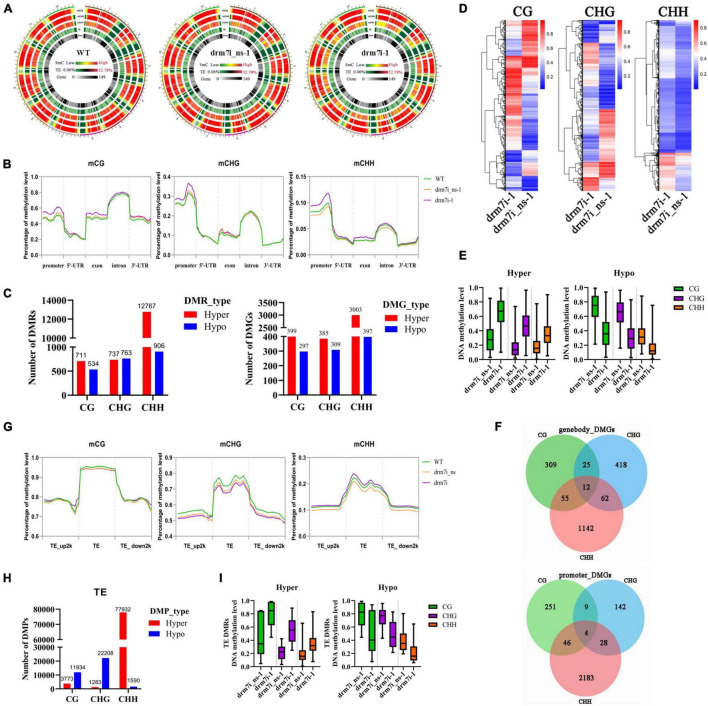
Effect of *SlDRM7*-RNAi on whole-genome DNA methylation. **(A)** Whole-genome bisulfite sequencing (WGBS) reveals DNA methylation profiles globally. Genome-wide methylations of 12 tomato chromosomes from WT, drm7i_ns-1, and drm7i-1 samples are illustrated in the *^m^*CG, *^m^*CHG, and *^m^*CHH contexts (where H is A, T, or C), and *^m^*C for TEs and genes. **(B)** Density plots of differential *^m^*CG, *^m^*CHG, and *^m^*CHH at the regions of the promoter, 5’-UTR, exon, intron, and 3’-UTR. Green, orange, and purple lines represent WT, drm7i_ns-1, and drm7i-1, respectively. **(C)** Numbers of differentially methylated regions (DMRs) and differentially methylated genes (DMGs) at CG, CHG, and CHH sites in drm7i vs. drm7i_ns. **(D)** Heat maps of the methylation level of DMRs at CG, CHG, and CHH sites in drm7i vs. drm7i_ns. **(E)** Overall DNA methylation level of hyper- and hypo-DMRs at CG, CHG, and CHH sites identified in drm7i_ns vs. drm7i. **(F)** Venn diagrams of DMGs associated with gene body (left) and promoter regions (right) in drm7i. **(G)** Density plots of differential mCG, mCHG, and mCHH at TEs and their 2-kb upstream and downstream regions. WT, drm7i_ns-1, and drm7i-1 are indicated. **(H)** Numbers of differentially methylated probes (DMPs) of TEs at CG, CHG, and CHH sites in drm7i-1 vs. drm7i_ns-1. **(I)** Methylation levels of hyper- and hypo-TEs in CG, CHG, and CHH sites.

To elucidate how *SlDRM7*-RNAi regulates the leaf interveinal chlorosis/senescence, whole-genome bisulfite sequencing (WGBS) was performed on the 2nd leaf collected from WT, drm7i_ns-1, and drm7i-1 seedlings at the six-leaf stage. We observed that *SlDRM7*-RNAi did not cause any significant alterations in genome-wide *^m^*C (Figure 4A; Supplementary Tables 2, 3). However, upon evaluating methylation levels within gene body or promoter regions, we found genome-wide hypermethylation at CHG, CG, and CHH sites of drm7i-1 compared to WT and drm7i_ns-1 (Figure 4B), resulting in the production of a number of differentially methylated regions (DMRs) and differentially methylated genes (DMGs) in drm7i-1. A similar scenario where the increased whole-genome methylation caused by *SlMET1* RNAi has been reported previously (Yao et al., 2020). Generally, most hypermethylated DMRs (hyper-DMRs) occurred at CHH sites, where *^m^*CHH-type hyper-DMRs/DMGs accounted for more than half (Figure 4C). Although *^m^*CHH-type DMRs were much more abundant than *^m^*CG-type or *^m^*CHG-type DRMs, their methylation levels were lowest and less influenced by *SlDRM7* silencing (Figures 4D,E). In addition, when assessing the DMRs associated with different gene features of DMGs, there were 12 DMGs possessing DMRs in all three contexts located at gene body, and 4 DMGs located at promoters, respectively (Figure 4F).

Previous studies have suggested that hypermethylation of transposable elements (TEs) is probably responsible for the suppression of active transposons (Nuthikattu et al., 2013; Yang et al., 2019). We further analyzed methylation levels within Tes and their flanking regions. In general, *^m^*CG, *^m^*CHG, and *^m^*CHH all show higher levels in TEs than in both 2-kb upstream and downstream regions in the tomato genome (Figure 4G), consistent with the methylation patterns in Arabidopsis and rice genomes (Le et al., 2014; Zhang et al., 2015). However, by contrast with WT and drm7i_ns-1, *^m^*CHG and *^m^*CG decreased while *^m^*CHH increased within TEs of drm7i-1 (Figure 4G). Moreover, 118,720 differentially methylated probes (DMPs) were identified, of which the majority were preferentially hypermethylated in the CHH context, although greater differences of methylation levels were observed in *^m^*CG and *^m^*CHG rather than that in *^m^*CHH (Figures 4H,I).

To understand the biological functions of DMGs, Gene Ontology (GO) enrichment and Kyoto Encyclopedia of Genes and Genomes (KEGG) pathway enrichment analyses were performed. The GO enrichment showed that the hyper-DMGs were significantly enriched in terms of “extracellular region” and “catalytic activity,” which contained 37 and 565 DMGs, respectively (Supplementary Figure 5A). The KEGG pathway enrichment revealed that abundant hyper-DMGs were mainly assigned into five pathways related to “metabolic pathways,” “biosynthesis of secondary metabolites”, “starch and sucrose metabolism,” “linoleic acid metabolism,” and “phenylpropanoid biosynthesis,” containing 166, 94, 26, 22, 6, and 22 DMGs, respectively (Supplementary Figure 5B). For hypo-DMGs, neither GO nor KEGG enriched any terms significantly (Supplementary Figures 5C,D).

**FIGURE 5:**
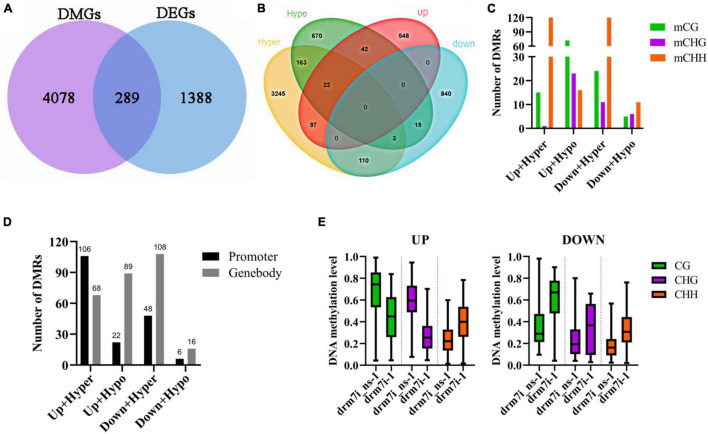
Combinational analysis of differential DNA methylated genes (DMGs) and differentially expressed genes (DEGs). **(A)** Venn diagrams of DMGs and DEGs (fold change > 2, FDR < 0.05, and drm7i-1 or drm7i_ns-1 FPKM > 1). **(B)** Venn diagrams of hypermethylated/hypomethylated genes and up-/downregulated genes. **(C)** Numbers of meth-DMGs in CG, CHG, and CHH contexts. **(D)** Numbers of meth-DMGs with DMRs located at promoters or gene bodies. **(E)** Overall DNA methylation levels of meth-DEGs in drm7i-1 vs. drm7i_ns-1.

To further investigate whether SlDRM7-mediated DMRs affect gene expression, RNA-seq was performed using the same leaf samples for WGBS. From the comparative “drm7i-1 vs. drm7i_ns-1” transcriptomes, 709 and 968 differentially expressed genes (DEGs) were identified to be up- and downregulated, respectively (Supplementary Figure 6A). However, there were only 289 unique DEGs with either *^m^*CG, *^m^*CHG, or *^m^*CHH-type DMRs (Figure 5A), which were designed as meth-DEGs thereafter. Among those meth-DEGs possessing both hyper- and hypo-DMRs, 22 were upregulated, while the other 3 are downregulated (Figure 5B). Most DMRs of meth-DEGs occurred in the CHH context, except that hypo-DMRs of upregulated meth-DEGs dominated in the CG context (Figure 5C). Indeed, hyper-DMRs of upregulated meth-DEGs preferentially occurred in promoters, while others were predominantly in the gene body (Figure 5D). Apart from *^m^*CHH with a moderate increase in drm7i-1, the methylation levels of upregulated meth-DEGs in CG and CHG contexts reduced 22 and 31%, respectively (Figure 5E). These results establish that different meth-DEGs may involve in different epi-regulatory modes mediated by silencing of *SlDRM7*. It is worthwhile noting that our comparative transcriptional profiling does not reveal any off-target effect on the expression of genes that may be related to *SlDRM7* in these RNAi lines.

### *SlDRM7* Epi-Controls Gene Expression Related to Photosynthesis and Chlorophyll Metabolism

**FIGURE 6:**
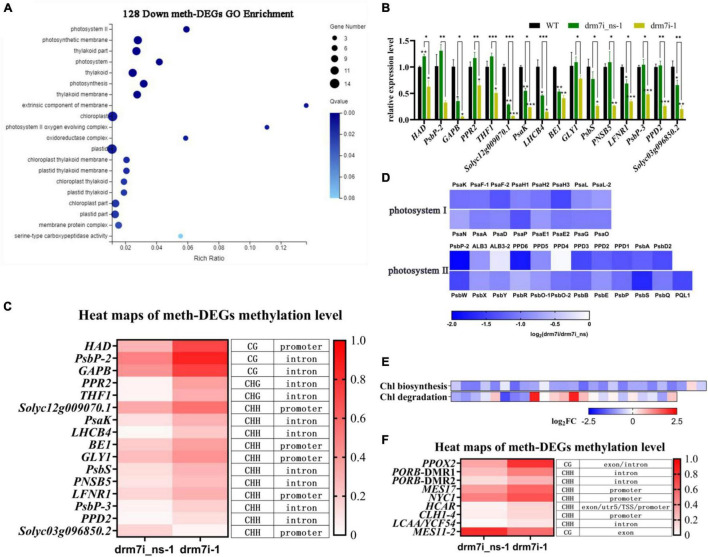
Analysis of down-regulated meth-DEGs. **(A)** Gene ontology enrichment analysis of 128 downregulated meth-DEGs. The size of the circle represents gene numbers, and the color represents the FDR. **(B)** Validation of RNA-seq using RT-qPCR for meth-DEGs involved in photosynthetic or chloroplast-related terms in **(A)**. Data are shown as means ± SD of three biological duplicates. Asterisks (above error bar represent the difference with WT; above the short line represent the difference with drm7i_ns) indicate the significant differences (*P ≤ 0.05, **P ≤ 0.01, ***P ≤ 0.001, one-way ANOVA, Tukey’s HSD). **(C)** Heat map illustration of the methylation level of meth-DEGs shown in **(B)**. **(D)** Heat map display of gene expression in the photosynthesis light reactions. Gene expression was determined by RNA-seq. **(E)** Heat map of expression of genes related to chlorophyll biosynthesis and chlorophyll degradation. Gene expression was determined by RNA-seq. **(F)** Heat map of the methylation levels of meth-DEGs in **(E)**.

To explore which biological processes the above meth-DEGs participate in, using GO enrichment analysis, the above 289 meth-DEGs were implied in “beta-glucosidase activity,” “photosystem II,” “glucosidase activity,” and “oxidoreductase activity” (Supplementary Figure 6B; Supplementary Table 4). Exhilaratingly, 128 downregulated meth-DEGs were significantly enriched in 20 putative pathways, most of which were linked to “chloroplast-related cellular component metabolism or photosynthesis” (Figure 6A; Supplementary Table 5), and 16 candidates out of them were further screened for RT-qPCR analysis. Consistent with RNA-seq data (Supplementary Table 5), the transcript levels of these genes were significantly depressed in drm7i-1 compared with drm7i_ns-1 or WT (Figure 6B). Apart from Solyc03g096850 that encodes a mitochondrial out membrane transporter protein presenting hypomethylation at CHH sites within its promoter, the rest of 15 meth-DEGs possessed hypermethylation at either CG, CHG, or CHH sites within promotors or gene body regions (Figure 6C).

It was known that *SlPSKA*, *SlLHCB4*, *SlPsbP*, *SlPsbP-2*, *SlPsbP-3*, and *Solyc10g047410.1* encode photosystem family proteins to form core complexes as well as light-harvesting complexes (LHCI and LHCII). The light reaction is catalyzed by two photosystems, photosystem I (PS I), and photosystem II (PS II), where the light energy is harvested to drive the transfer of electrons from water, *via* a series of electron donors and acceptors, to the final acceptor NADP^+^, which is finally reduced to NADPH (Nelson and Yocum, 2006; Jarvi et al., 2015). By multiple sequences alignment with those encoding core complexes of light reactions in Arabidopsis, a total of 88 tomato orthologous were identified, of which 42 genes were expressed in leaves (WT FPKM > 1) (Supplementary Table 6). Compared with drm7i_ns-1, the transcriptional level of 19 genes in drm7i-1 was significantly inhibited (Figure 6D; Supplementary Table 6). Since a set of LHCI and LHCII subunits binds to the core complex to capture the light and to bound chlorophylls (Li et al., 2000; Scheller et al., 2001; Jarvi et al., 2015), we also found 32 genes encoding LHC proteins in tomato. Compared with WT, the expression of these LHC genes were significantly repressed in drm7i-1, except for *Solyc05g056080.2* and *Solyc05g056060.2* (Supplementary Figure 6C). Moreover, 3 out of 32 genes, i.e., *Solyc08g067320.1*, *Solyc07g063600.2*, and *Solyc09g014520.2*, were meth-DEGs with *^m^*CHH-type hyper-DMRs located at promotor or intron regions (Supplementary Figure 6D).

Considering that chlorophyll content decreased in drm7i leaves (Figure 2B, left panel), we further investigated whether candidates involved in chlorophyll biosynthesis and/or degradation could be identified as a meth-DEG. By screening a tomato orthologous that is linked to chlorophyll metabolism as previously indicated in Arabidopsis (Kobayashi and Masuda, 2016; Lin et al., 2016) (Supplementary Table 7), expression levels of most genes involved in chlorophyll biosynthesis were found to be significantly decreased in drm7i-1 (Figure 6E; Supplementary Figure 6E), where SlPPOX2 and SlPORB were meth-DEGs (Figure 6F). On the other hand, another 6 meth-DEGs were observed to be related to chlorophyll degradation (Figure 6F). Nevertheless, unlike DEGs associated with chlorophyll biosynthesis, no obvious variation trend was observed in the expression of DEGs linked to chlorophyll degradation, among which some were unchanged, some were downregulated, and some were upregulated (Figure 6E; Supplementary Table 7). These findings imply that the inhibition of chlorophyll biosynthesis, rather than acceleration of chlorophyll degradation, might be important to induce chlorophyll loss, leading to the subsequent formation of chlorosis in drm7i leaves.

### Silencing of SlDRM7 Blocked Starch Degradation and Caused Chloroplast Dysfunction

**FIGURE 7:**
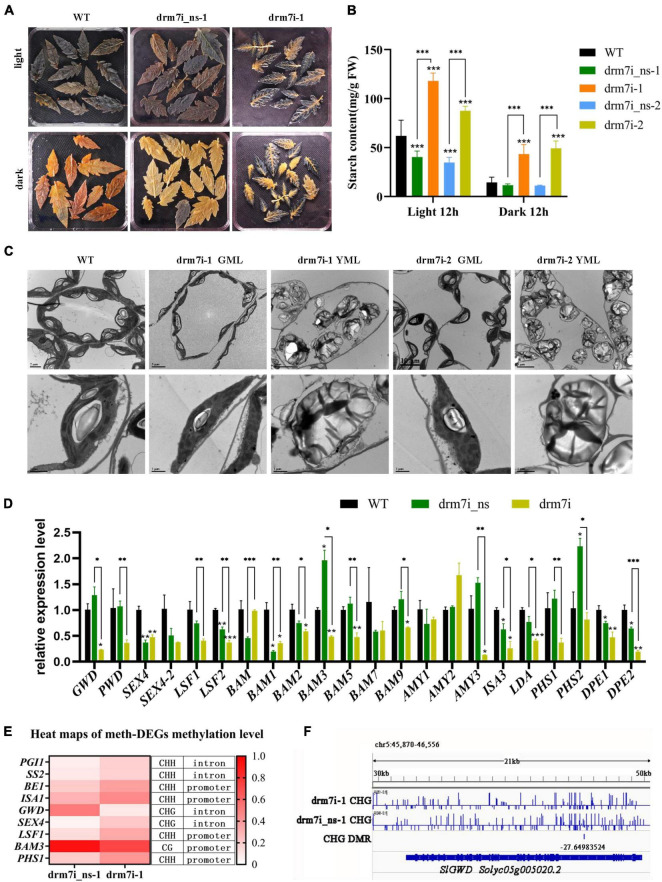
SlDRM7 functions in starch metabolism. **(A,B)** Iodine-iodide kalium staining **(A)** and measurement **(B)** of starch content of the leaves in a 12-h photoperiod. By harvesting at the end of daylight (light 12 h) and the end of the night (dark 12 h), leaves at the same position of the drm7i_ns-1 and drm7i-1 lines at the six-leaf stage were stained with Lugol’s solution to visualize starch, and then the starch content was measured physiologically. **(C)** TEM examination of chloroplast structures. Leaf mesophyll cells were collected from the 2nd leaves of AC, two *SlDRM7*-RNAi lines drm7i-1 and drm7i-2 at the six-leaf stage. The Upper and lower panels show a group of or individual chloroplasts. Bars are inserted as indicated. S, starch granule; OG, osmiophilic plastoglobuli; GL, Grana lamella; SL, stroma lamella. **(D)** Relative expression of genes related to starch degradation in WT, drm7i_ns-1, and drm7i-1. Data are represented as means ± SD of three biological duplicates. Asterisks (above error bar represent the difference with WT; above the short line represent the difference with drm7i_ns) indicate the significant differences (*P ≤ 0.05, ^**^P ≤ 0.01, ^***^P ≤ 0.001, one-way ANOVA, Tukey’s HSD). **(E)** Heat map of the methylation levels of meth-DEGs involved in starch metabolism. **(F)** Detailed DNA methylation profile at the *SlGWD* (*Solyc05g005020.2*) loci. Differential DNA methylation is seen in the drm7i vs. drm7i_ns. A screenshot from the Integrative Genomics Viewer (IGV) display of whole-genome bisulfite sequencing data is shown. Vertical bars on each track indicate DNA methylation levels. Red boxes represent the DMRs.

The comparative transcriptomes revealed that 59 unique DEGs in drm7i lines were linked to terms of “starch and sucrose metabolism,” the most significant category in the KEGG enrichment pathway (Supplementary Figure 7A). Starch is the transient storage photosynthate in many higher plants. During rapid growth, starch accumulates in the chloroplast in the daytime and degrades at night, thereby providing a steady supply of carbohydrates for organs to sustain metabolism and growth (Zeeman et al., 2002). Defects in starch turnover have been shown to impact plant growth in various species (Eimert et al., 1995; Corbesier et al., 1998; Harrison et al., 2000; Yu et al., 2001; Nashilevitz et al., 2009; Vriet et al., 2010). Therefore, we tracked diurnal variation of starch in leaves evidenced by Lugol’s staining. Notably, after growing in daylight for 12 h, WT, drm7i_ns-1, and dim7i-1 exhibited bright blue-purple color (Figure 7A, upper panel); however, after undergoing in dark for 12 h, the staining color of starch almost completely disappeared in WT and drm7i_ns-1, while remained substantially in drm7i-1, especially in leaf lamina where chlorosis developed (Figure 7A, bottom panel). This finding suggested that, in drm7i-1 leaves, starch degradation occurred in green regions near the vein but not in interveinal yellowing regions. Consistently, after 12-h treatment of daylight or dark, starch content in drm7i was significantly higher than that in WT or drm7i_ns, indicating that the starch degradation was inhibited in drm7i leaves (Figure 7B).

In severe *starch excess* (*sex*) mutant, whole-chloroplast degradation is due to the highly accumulated starch caused by an imbalance between the daytime excessive starch synthesis and night-time limited breakdown (Stettler et al., 2009), which prompted us to examine the cellular ultrastructure of mesophyll cells in drm7i-1 leaves using transmission electron microscopy (TEM). As shown in Figure 7C, when compared with the chloroplasts in the leaves of WT, chloroplasts in GLM of drm7i lines resembled WT, but often contained grana lamella with a slightly disorganized appearance. However, it was noteworthy that in YLM of drm7i lines, highly misshapen chloroplasts were observed, in which grana and stroma thylakoids were completely disrupted, leaving an aberrant accretion of starch granules that appeared as swollen. Interestingly, the chloroplast degradation that occurred in YLM of drm7i lines was distinguishably different from that occurred in naturally senescent WT leaves, where thylakoid membrane system and starch granules were normally degraded (Supplementary Figure 7B), suggesting a linkage between starch accumulation and chloroplast homeostasis in *SlDRM7*-mediated leaf chlorosis/senescence. Taken together, our findings demonstrate that silencing of *SlDRM7* led to an excessive accumulation of starch in chloroplasts that causes chloroplast dysfunction, resulting in tomato leaf chlorosis/senescence, a similar phenotype in *sex* mutants.

To elucidate the possible cause(s) underlying the *sex-like* phenotype of drm7i lines, genes related to starch metabolism and sugar transporter were chosen from the tomato genome (Supplementary Table 8). The RT-qPCR analysis showed that, compared with WT and drm7i_ns-1, some starch biosynthesis-associated genes were downregulated, but none was upregulated in drm7i-1 (Supplementary Figure 7C), despite 4 of them were hyper-DMGs at CHH sites (Figure 7E), indicating that the *sex-like* phenotype of drm7i lines did not result from the acceleration of starch biosynthesis. Besides, expression of most starch degradation-associated genes was repressed in drm7i-1 (Figure 7D), including 4 meth-DEGs, i.e., *SlGWD, SlLSF1, SlBAM3*, and *SlPHS1*, which encode glucan water dikinase, phosphoglucan phosphatase, beta-amylase-3, and alpha-1, 4 glucan phosphorylase isozyme, respectively. The methylation levels of *SlGWD* and *SlBAM3* were decreased by 42.6% in the CHG context located at intron regions, and 20.4% in CG context located at promoter regions, respectively (Figure 7F). Both *SlLSF1* and *SlPHS1* had hypermethylation at CHH sites (approximately 20%) within promoter regions (Figure 7E). It is worth noting that SlGWD is a key enzyme in controlling the phosphate content of starch and that Arabidopsis *gwd* mutant exhibits the most severe *sex* phenotype (Yu et al., 2001). These data suggested that the suppression of starch degradation is a key factor for starch accumulation and is closely linked with leaf chlorosis mediated by *SlDRM7*-silencing-induced epi-regulation.

In addition, we found a decreased expression of three plastidic phosphate translocator genes and two sugar transporter genes in drm7i-1 vs. WT or drm7i_ns-1, including glucose-6-phosphate/phosphate translocator (SlGPT1), phosphoenolpyruvate/phosphate translocator (SlPPT2), triose-phosphate/phosphate translocator (SlTPT), maltose transporter (SlMEX1), and glucose transporter (SlGLT1). These data suggested that the translocation between phosphate and photosynthates was repressed, resulting in growth retardation of drm7i lines (Supplementary Figure 7D). However, none of these genes possesses obvious DMRs, thereby, their expression may be unlikely affected directly by *SlDRM7*-mediated epigenetic control.

## Discussion

### *SlDRM7* Impacts Tomato Methylome

Plant DRMs are not only required for *de novo* methylation but also act with CMT3 to maintain non-CG methylation (Cao and Jacobsen, 2002a; Cao et al., 2003). For instance, rice OsDRM2, Arabidopsis DRM2, and tobacco (*Nicotiana tabacum*) NtDRM1 have *de novo* methylation activity and a preference for non-CG methylation (Cao et al., 2003; Wada et al., 2003; Moritoh et al., 2012; Gouil and Baulcombe, 2016). Surprisingly, although no obvious changes in overall genome-wide methylation level were caused by *SlDRM7*-RNAi (Figure 4A), methylation levels of different gene features in drm7i lines, especially at promoter and exon regions, were different from that in WT and drm7i_ns lines (Figure 4B). In addition, the hypermethylation and hypomethylation levels of DMRs were equivalent (Figures 4E,I), which might be accountable for no significant alterations in whole-genome *^m^*C. While the majority of DMRs were preferentially hypermethylated in the CHH context, the magnitude of differential methylation was much greater for CG and CHG contexts (Figure 4D). Similarly, increased CHH methylation was observed in the Arabidopsis *met1* mutant (Mathieu et al., 2007). One possible explanation is that CHH methylation might be a result of changes in DMLs expression that was affected by *SlDRM7* (Supplementary Figure 8). It has been reported that CHH hypermethylation occurred when DNA demethylase (DMLs) was repressed (Liu and Lang, 2020).

### *SlDRM7* Affects Plant Growth and Development

We reveal that SlDRM7 is required for proper plant growth and development and, more importantly, for preventing leaf chlorosis and premature senescence (Figure 1; Supplementary Figures 1, 3). Although the function of genes involved in *de novo* DNA methylation and maintenance in plant growth and development have been reported previously, the interveinal chlorosis exhibited in tomato drm7i lines was never observed in other plant species. For example, rice *osdrm2* disruption displayed pleiotropic developmental defects at both vegetative and reproductive stages including semi-dwarfed stature, reductions in tiller number, abnormal panicle, and spikelet morphology (Moritoh et al., 2012). Maize ortholog of DRM2 loss-of-function lines, *dmt103*, had developmental defects in the reproductive stage but no morphological phenotypes (Garcia-Aguilar et al., 2010). However, Arabidopsis *drm1 drm2* mutant had WT phenotypes (Cao and Jacobsen, 2002b). The different effects of DRMs on plant development in different species may be ascribed to the distribution of repeats and TEs and different genome sizes or the abundance (He et al., 2011). We observed a higher degree of methylation within the TEs enrichment region than a gene body in the tomato genome (Figure 4G). Compared to Arabidopsis, crop genomes such as tomato, rice, and maize have not only more abundant TEs but also more genes with highly methylated TEs (Lang et al., 2017). In addition, given the more complex genome (∼900 Mb) of tomato in comparison with Arabidopsis genome (∼125 Mb) or rice (∼370 Mb), it is possible that the tomato SlDRMs play more intricate functions in growth and development.

### *SlDRM7*-Mediated Epi-Control Influences Starch Metabolic Pathways

First, RNA-seq showed that a considerable proportion of genes related to starch degradation were downregulated in drm7i lines compared to drm7i_ns and WT plants (Figure 7D). Second, several genes were identified as meth-DEGs including *SlGWD*, suggesting that these genes could be regulated through SlDRM7-induced epi-control. In Arabidopsis, GWD was reported to be involved in the initiation of starch degradation, since *gwd* mutants had more severe *sex* phenotypes than mutations affecting steps downstream of GWD (Critchley et al., 2001; Lu and Sharkey, 2004). In the absence of GWD activity, starch degradation was impaired leading to a severe *sex* phenotype not only in Arabidopsis but also in potato (*Solanum tuberosum*) and *Lotus japonicas* (Lorberth et al., 1998; Yu et al., 2001; Nashilevitz et al., 2009; Garcia-Aguilar et al., 2010). Here, TEM analysis demonstrated that silencing of *SlDRM7* resulted in excessive accumulation of starch granules in chloroplasts, which triggered severe *sex* phenotype in tomato leaves as well (Figure 7C). Additionally, pollen in tomato *gwd* mutant with a *sex* phenotype was associated with a reduction in pollen germination and caused a male gametophytic lethality (Nashilevitz et al., 2009), indicating that the deficiency of SlGWD affects both the vegetative and reproductive growth in tomatoes. Moreover, *SlPWD* and other genes acting downstream of SlGWD contributing to starch degradation were also suppressed in drm7i (Figure 7D), reflecting the key position of SlGWD to initiate the biodegradation of starch.

We further found that starch accumulation was accompanied by chloroplasts dysfunction in *drm7i* lines, indicating that starch metabolism is critical for chloroplast homeostasis (Figure 7C). In agreement with our supposition, Arabidopsis *maltose excess 1* (*mex1*) mutant accumulated high levels of starch in chloroplasts and displays autophagy-like chloroplast degradation (Stettler et al., 2009). However, leaf senescence and senescence-related chlorophyll catabolism are not induced in *mex1* (Stettler et al., 2009), whereas our results showed silencing of *SlDRM7* reduced chlorophyll content and repressed expression of genes involved in the chlorophyll biosynthesis and subsequently induced leaf senescence (Figures 2, 7B). These findings indicated that, apart from starch metabolism, SlDRM7-induced epi-control has a wide range of effects on plant growth and development in tomatoes.

### SlDRM7 Modulates Leaf Chlorosis and Senescence

Leaf chlorosis and senescence involve complex genetic programming and less understood epigenetic re-programming (Gan, 2003; Guo and Gan, 2005; Ay et al., 2014; Woo et al., 2019). In comparison to drm7i_ns-1, we found 289 genes, such as *SlGWD*, in the whole genome were meth-DEGs in drm7i-1 (Figure 5A). Whether SlDRM7 directly affects methylation levels at these specific sites or indirectly through other processes remains unknown. Also, it is unclear whether DNA methylation changes are directly related to gene expression regulation. In some cases, DNA methylation changes appear to be associated closely with the transcriptional control of specific loci in plants (Kinoshita and Seki, 2014; Yong-Villalobos et al., 2015; Feng et al., 2016). Here, we found that intron hypomethylation of *SlGWD* is related to the expression repression in *SlDRM7i* lines (Figure 7E). Gene body methylation can be related to transcriptional upregulation and has been suggested to protect genes from aberrant transcription caused by cryptic promoters (Zhang et al., 2006; Feng et al., 2016). On the other hand, a poor correlation between DNA methylation and gene expression has also been reported in Arabidopsis (Meng et al., 2016; Yen et al., 2017; Chen et al., 2018c; Fan et al., 2020). Indeed, there are a large number of DEGs including *SAGs*, TFs related to senescence, and genes involved in the photosynthetic system, chlorophyll metabolism, and Calvin cycle, whose methylation levels remain unchanged in *SlDRM7i* lines (Figure 3; Supplementary Tables 6–9).

NAC family proteins are found to act as regulators during leaf senescence (Woo et al., 2019), and the orthologs of Arabidopsis ORESARA1 (AtORE1), namely SlORE1S02, SlORE1S03, and SlORE1S06, have received special attention for encoding a master regulator of senescence initiation, which can induce senescence-related genes *SlSAG12* and interact physically with and inactivates the chloroplast maintenance-related TF SlGLK1 (Garapati et al., 2015; Lira et al., 2017). The SlNAP2 has a complex role in establishing ABA homeostasis during leaf senescence (Ma et al., 2018; Ma X. et al., 2019). The decrease of Calvin cycle activity in chloroplasts leads to an increased generation of reactive oxygen species (ROS) in the respective organelle, which worked as signaling molecules involved in the regulation of senescence (Navabpour et al., 2003). Therefore, the pleiotropic phenotype in drm7i lines may be affected by multiple pathways, and there might be the indirect effects of *SlDRM7*-mediated epi-control on regulating the expression of many development-related genes.

**FIGURE 8:**
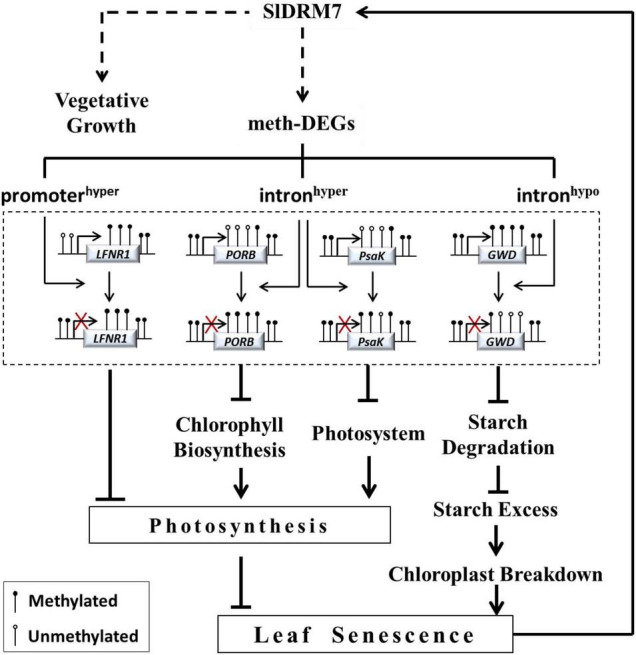
A model for SlDRM7 epi-controls leaf chlorosis and senescence. SlDRM7-mediated DMRs affect gene expression, which is designed as meth-DEGs. Silencing of *SlDRM7* influences DNA methylation in promoter and gene body, and leads to transcriptional inhibition of genes directly or indirectly as exemplified by *SlLFNR1*, *SlPORB*, *SlPsaK*, and *SlGWD*. We hypothesized that these changes including promoter hypermethylation of *SlLFNR1*, intron hypermethylation of *SlPORB* and *SlPsaK*, and intron hypomethylation of *SlGWD* resulted in their expression repression, which inhibited photosynthesis and starch degradation, eventually leading to leaf chlorosis and senescence. Conversely, leaf senescence can induce *SlDRM7*, forming a feedback regulatory loop, to balance vegetative growth and senescence.

It is found that global DNA demethylation occurs during Arabidopsis leaf senescence (Ogneva et al., 2016). However, *SlDRM7* expression was induced in both natural senescence and dark-induced aging (Supplementary Figure 4). Therefore, tomatoes might have a feedback regulation mechanism to inhibit leaf senescence by increasing *SlDRM7* expression and consequently affecting genome-wide DNA methylation levels, and *SlDRM7* seems to be a necessary anti-senescence factor during the growth and development in tomatoes. Based on our results, we propose a regulatory model to illustrate the relationship between *SlDRM7*-mediated epi-control and leaf senescence (Figure 8). The *SlDRM7* functions as an epi-regulator to modulate the expression of meth-DEGs to affect leaf development and senescence. Silencing of *SlDRM7* leads to hypermethylation or hypomethylation within promoter or intron regions, which directly or indirectly suppresses a set of meth-DEGs involved in photosynthesis, chlorophyll biosynthesis, photosystem, and starch degradation. Subsequently, the inhibition of these genes reduces chlorophyll content and photosynthetic capacity and triggers chloroplast breakdown as well, which resulted in leaf chlorosis and senescence. Meanwhile, an unknown self-feedback regulatory pathway is established by activating *SlDRM7* expression to make a balance between vegetative growth and senescence.

## Data Availability Statement

The original contributions presented in the study are publicly available. This data can be found here: All tomato materials, including wild-type *Solanum lycopersicum* cv. Ailsa Craig (AC), the *SlDRM7*-RNAi lines and *SlDRM7*-KO lines (AC background), are deposited in our lab. Sequence data can be found in NCBI (https://www.ncbi.nlm.nih.gov/) and Phytozome (https://phytozome-next.jgi.doe.gov/), two comparative platform for green plant genomics. Gene ID was shown in Supplementary Tables 1, 4–8. WGBS and RNA-seq data sets have been deposited in NCBI under Bioproject ID: PRJNA773102 and PRJNA772527, respectively.

## Author Contributions

YW, WC, and JY designed and performed the experiments, analyzed the data, and wrote the manuscript. YW and LH performed the bioinformatics analyses. HZ, JW, GH, and RH cultured the plants and performed the experiments. SZ was involved in data analysis and helped writing the manuscript. YH, JY, and WC initiated the project, conceived the experiments, analyzed the data, and wrote the manuscript. All authors edited and finalized the manuscript.

## Conflict of Interest

The authors declare that the research was conducted in the absence of any commercial or financial relationships that could be construed as a potential conflict of interest.

## Publisher’s Note

All claims expressed in this article are solely those of the authors and do not necessarily represent those of their affiliated organizations, or those of the publisher, the editors and the reviewers. Any product that may be evaluated in this article, or claim that may be made by its manufacturer, is not guaranteed or endorsed by the publisher.

## Footnotes

1
https://solgenomics.net

2
http://ftp.ensemblgenomes.org/pub/plants/current/fasta/solanum_lycopersicum/dna/

3
http://www.geneontology.org/

4
https://www.kegg.jp/

5
https://en.wikipedia.org/wiki/Hypergeometric_distribution
