# Supplementary material for: Potential Role of Domains Rearranged Methyltransferase7 in Starch and Chlorophyll Metabolism to Regulate Leaf Senescence in Tomato
Source: Front Plant Sci. 2022 Feb 8;13:836015. doi: 10.3389/fpls.2022.836015 (PMC8860812; doi:10.3389/fpls.2022.836015)
Supplement: Supplementary file 1 [file Data_Sheet_1.docx]

## Supplementary Figure S1

Supplementary Figure 1. Phenotypes of WT, *SlDRM7*-RNAi and *SlDRM7*-knockout transgenic lines. (A) Leaf development and chlorosis. Wild-type AC (WT), transgenic drm7i_ns-1 and drm7i-1 seeds were germinated on plates containing 1/2 MS medium for 6 days. Plantlets were transferred to 1/5 Hoagland solution and grown for 4 weeks, subsequently to soil for growth and seed collection. Photographs were taken at the end of 1, 2, 3 and 4 weeks (w) after transferring to hydroponic culture, and 5 and 9 weeks after transferring to soil. Bars = 5cm. (B) CRISPER/CAS9 gene editing of *SlDRM7*-knockout (KO) mutant. Gene structure of *SlDRM7* and two sgRNA for targets are shown. Sequence alignments of two KO lines (KO-1 and KO-2) with the reference are shown. (C-H) Phenotype of drm7i-1 and KO plants at the same stage. drm7-1 maintains interveinal chlorosis / senescence leaves (C), the old leaves showed more severe phenotypes compared to the young leaves (F), while young and old leaves of KO-1 (D, G) and KO-2 (E, H) displayed normal wild-type (WT) green leaves. Tomato seeds were spread and germinated directly in compost, seedlings were photographed at 9 weeks after germination in C to E with bars = 5 cm. Young and old leaves collected from drm7i-1 and two KO lines were photographed in panel (F) to panel (H) with bars = 2 cm.

## Supplementary Figure S2

Supplementary Figure 2. Effect of *SlDRM7* knockout on the expression of other three *SlDMR* genes. Data are means ± SD of three biological replicates. *ACTIN* was used as the internal control. Asterisks indicate the significant differences compared with WT (*P ≤ 0.05, *** P ≤ 0.001, one-way ANOVA, Tukey’s HSD).

## Supplementary Figure S3

Supplementary Figure 3. Morphological traits of WT and *SlDRM7*-RNAi lines. (A) Leaf development is shown by leaf size and chlorosis in the WT, two drm7i_ns lines, and two drm7i lines at six-leaf stage. (B, C) Leaf size of the WT, two drm7i_ns lines, and two drm7i lines, respectively. Average leaf areas were measured for 1^st^, 2^nd^ and 3^rd^ leaf from WT, drm7i_ns-1 and drm7i-1 (B); and for that of 1^st^, 2^nd^ and 3^rd^ leaf from WT, drm7i_ns-2 and drm7i-2 (C) at six-leaf stage. (D) Morphological phenotypes with shoot of WT, two drm7i_ns lines, and two drm7i lines at six-leaf stage. (E, F) the shoot height of WT, drm7i_ns-1 and drm7i-1 (E); and WT, drm7i_ns-2 and drm7i-2 (F) at 1, 2, 3, 4 weeks after transferring to 1/5 Hoagland solution. Data are shown as means ± SD of ten biological replicates. The error bar represents the standard error of SD. Asterisks (above error bar represent the difference with WT; above the short line represent the difference with drm7i_ns) indicate the significant differences (* P ≤ 0.05, ** P ≤ 0.01, *** P ≤ 0.001, one-way ANOVA, Tukey’s HSD).

## Supplementary Figure S4

Supplementary Figure 4. Natural senescence and dark-induced senescence induce *SlDRM7* expression. (A) RT-qPCR analysis of the transcript level of *SlDRM7* in the young leaf (YL) and natural senescence leaf (SL) at 1-month stage of tomato wild-type plants. (B) RT-qPCR analysis of the mRNA level of *SlDRM7* in light control and dark-treated seedlings at different developmental stages of tomato wild-type plants. Total RNA for RT-qPCR was extracted from AC cotyledons, 2-leaf-stage and 4-leaf-stage AC seedlings grown in dark (Dark) or under normal light (Light) for 7 days. Data are shown as means ± SD of three biological replicates. Relative *SlDRM7* expression level was normalized against *ACTIN* mRNA. One-way ANOVA (Tukey’s HSD) statistical analysis was performed for YL vs SL, or Light vs Dark (** P ≤ 0.01, *** P ≤ 0.001).

## Supplementary Figure S5

Supplementary Figure 5. Biological functions of DMGs. (A) Two enriched Gene Ontology (GO) terms (P < 0.05) of hyper-DMGs. (B) KEGG pathway enrichment analysis of hyper-DMGs. The size of the circle represents gene numbers, and the color represents the FDR. (C) GO enrichment analysis of hypo-DMGs. (D) KEGG pathway enrichment analysis of hypo-DMGs. The size of the circle represents gene numbers, and the color represents the q-value.

## Supplementary Figure S6

**Supplementary Figure 6.** SlDRM7-induced DNA methylation correlated with the transcriptional regulation. **(A)** Numbers and volcano plot of differential expressed genes (DEGs) in drm7i-1 vs drm7i_ns-1 as determined by RNA-seq. Differentially expressed genes were defined as those with |Log_2_(FC) > 1|, FDR < 0.05 and one of drm7i-1 or drm7i_ns-1 FPKM >1. **(B)** GO enrichment analysis of 289 meth-DEGs. The size of the circle represents gene numbers, and the color represents the FDR. **(C)** Heat map showing the expression level of genes encoding light-harvesting complex (LHC) protein as determined by RNA-seq. The expression value is represented by log_2_(FC). **(D)** Heat map of the methylation levels of meth-DEGs which encode LHC protein. **(E)** Validation of RNA-seq using RT-qPCR for genes associated with chlorophyll biosynthesis. Data are shown as means ± SD of three biological replicates. Asterisks (above error bar represent the difference with WT; above the short line represent the difference with drm7i_ns-1) indicate the significant differences (* P ≤ 0.05, ** P ≤ 0.01, *** P ≤ 0.001, one-way ANOVA, Tukey’s HSD).

## Supplementary Figure S7

**Supplementary Figure 7.** *SlDRM7*-RNAi influenced starch metabolism. **(A)** KEGG pathway enrichment analysis of DEGs (fold change >2, FDR <0.05, and one of drm7i-1 or drm7i_ns-1 FPKM >1) in drm7i-1 vs drm7i_ns-1. The size of the circle represents gene numbers, and the color represents the FDR. **(B)** TEM micrographs of the chloroplast morphology in natural senescence leaves of WT (WT_OLD). Bars = 0.5 μm. S, starch granule; OG, osmiophilic plastoglobuli; GL, Grana lamella; SL, stroma lamella. **(C, D)** Relative expression of genes associated with starch biosynthesis (C) and sucrose transporter (D) in WT, drm7i_ns-1 and drm7i-1. Data are represented as means ± SD of three biological replicates. Asterisks (above error bar represent the difference with WT; above the short line represent the difference with drm7i_ns-1) indicate the significant differences (* P ≤ 0.05, ** P ≤ 0.01, *** P ≤ 0.001, one-way ANOVA, Tukey’s HSD).

## Supplementary Figure S8

Supplementary Figure 8. Expression analysis using RT-qPCR of *SlDMLs* in WT and *SlDRM7*-RNAi lines. (A-D) Relative expression of *SlDML1* (A), *SlDML2* (B), *SlDML3* (C) and *SlDML4* (D) in WT, drm7i_ns and drm7i transgenic lines. *ACTIN* was used as the internal control. Data are shown as means ± SD of three biological. Asterisks indicate the significant differences with WT (* P < 0.05, ** P < 0.01, *** P < 0.001, one-way ANOVA, Tukey’s HSD).
