# Supplementary material for: Potential Role of Domains Rearranged Methyltransferase7 in Starch and Chlorophyll Metabolism to Regulate Leaf Senescence in Tomato
Source: Front Plant Sci. 2022 Feb 8;13:836015. doi: 10.3389/fpls.2022.836015 (PMC8860812; doi:10.3389/fpls.2022.836015)
Supplement: Supplementary file 2 [file Table_1.DOCX]

**Supplemental Table S1. Primers used in this study.**

| Application | Gene name | Locus | Primer name | Primer sequence (5'-3') |
| --- | --- | --- | --- | --- |
| Primers used for constructing pRNAi-SlDRM7 | SlDRM7 | Solyc04g005250.2 | pSlDRM7i-F | CGACGACAAGACCCTGTTTACAATGAGAAGT |
|  |  |  | pSlDRM7i-R | GAGGAGAAGAGCCCTTATGTTCGGGTAGTGGA |
| Primers used for constructing SlDRM7-KO induced by CRISPR-CAS9 | SlDRM7 | Solyc04g005250.2 | sgRNA oligo-F | ATATATGGTCTCGATTGAGACAATGACAGCATTGACTGTT |
|  |  |  | sgRNA oligo-R | AACGGAATGAACTTAGATTGACACAATCTCTTAGTCGACTCTAC |
|  |  |  | sgRNA oligo-F1 | TGAGACAATGACAGCATTGACTGTTTTAGAGCTAGAAATAGC |
|  |  |  | sgRNA oligo-R1 | ATTATTGGTCTCTAAACGGAATGAACTTAGATTGACAC |
| Primers used for RT-qPCR | SlDRM7 | Solyc04g005250.2 | SlDRM7-F | CCAAGACGTCTCCGTTCAGC |
|  |  |  | SlDRM7-R | GAACAATGTGGTCGCCGTAG |
|  | SlSAG12 | Solyc02g076970.1 | SlSAG12-F | ACATCTCGCGAGTTGAACGA |
|  |  |  | SlSAG12-R | AAAATGCCCAGCAACATCCG |
|  | SlSAG13 | Solyc03g096640.2 | SlSAG13-F | CTCAACGAACGCTTACAAGAGTG |
|  |  |  | SlSAG13-R | GGGCTTCCTAATGTTAGTTCCCA |
|  | SlSAG15 | Solyc03g117950.2 | SlSAG15-F | GGAGTTGGAAAATCTGAACTGGC |
|  |  |  | SlSAG15-R | CGGATAGCTTCAGTTAGAGTCCC |
|  | SlSRG1 | Solyc02g071430.2 | SlSRG1-F | CCTGATGCCTTTGTTGTCAACAT |
|  |  |  | SlSRG1-R | TAGGAGCTGGACCTAAATCTCCA |
|  | SlGLK1 | Solyc07g053630.2 | SlGLK1-F | TGGGAATTGATTGTCTCACTCGT |
|  |  |  | SlGLK1-R | ATTCATCTCTCTCTTTCCTCCGC |
|  | SlORE1S03 | Solyc03g115850.2 | SlORE1S03-F | ACATTTCAGGGCTTGTGAGA |
|  |  |  | SlORE1S03-R | AGGTGAATTGTTGAAGGAATTGAT |
|  | SlORE1S06 | Solyc06g069710.2 | SlORE1S06-F | GATTCTGCTACTGCTACTGCTT |
|  |  |  | SlORE1S06-R | GGATCTTGAACCCCAAATGAAG |
|  | SlNAP2 | Solyc04g005610.2 | SlNAP2-F | GTGTCTGGTTATTGGAAAGCCAC |
|  |  |  | SlNAP2-R | GATCCATTTGGCCTAGTTGGTTG |
|  | HAD | Solyc02g069410.2 | HAD-F | TGCTGATGTACCTGTGCACA |
|  |  |  | HAD-R | TGCACCACCAAATCCATCCA |
|  | PsbP-2 | Solyc03g114930.2 | PsbP-2-F | AACAATTGTCCCGTTGCTGC |
|  |  |  | PsbP-2-R | TGGCGAACCAAAATCCCGTA |
|  | GAPB | Solyc12g094640.1 | GAPB-F | ACTCTCACGAGGTTGCCAAT |
|  |  |  | GAPB-R | TCAAGTCACGGTGTGATGCA |
|  | PPR2 | Solyc12g098990.1 | PPR2-F | CAACAACGTCATCGAAGCGT |
|  |  |  | PPR2-R | TGGACAGCGTTGATCACCTT |
|  | THF1 | Solyc07g054820.2 | THF1-F | ACTCAATCGGCGGAAAGGAA |
|  |  |  | THF1-R | AGCTGAACTGGACGTGCAAT |
|  |  | Solyc12g009070.1 | 009070-F | ACAAACCCTCAGCAGCTTGT |
|  |  |  | 009070-R | AGCCATTGCCAATTAAGCCT |
|  | PsaK | Solyc08g006930.2 | PsaK-F | GCTTGGTGGCAATTCAACCA |
|  |  |  | PsaK-R | TTTAGCCCTGCAGTTGCCTT |
|  | LHCB4 | Solyc09g014520.2 | LHCB4-F | ACATCCAATTCGCCGATCGA |
|  |  |  | LHCB4-R | CCTTTTTCGGTGCGGCTTTT |
|  | BE1 | Solyc07g064830.2 | BE1-F | TTCGCATGAAACAGCCTCCT |
|  |  |  | BE1-R | AATTGGCCCATGCAGGAACT |
|  | GLY1 | Solyc06g048920.2 | GLY1-F | AAACCCTTGTACTGCCGCTT |
|  |  |  | GLY1-R | GCTGCAATTGCTGTTCCGAA |
|  | PsbS | Solyc06g060340.2 | PsbS-F | TTGCTGCCTCTTTGTTGGGA |
|  |  |  | PsbS-R | AAGGATTTGCCAGGAGGGAT |
|  | PNSB5 | Solyc09g083190.2 | PNSB5-F | TCCTGTTGATCGATGGAGAACT |
|  |  |  | PNSB5-R | AAATCCTGTTGGGGGTGCAA |
|  | LFNR1 | Solyc02g083810.2 | LFNR1-F | TTGATGCCAATGGGAAGCCT |
|  |  |  | LFNR1-R | GGCATGAGCATTTCTTTGCCT |
|  | PsbP-3 | Solyc08g043180.2 | PsbP-3-F | CACCCACCAAAACCTTTGCA |
|  |  |  | PsbP-3-R | AGAAAAGCCGTCGCCATAGA |
|  | PPD2 | Solyc04g009420.2 | PPD2-F | GCTTCGCTATTGCCATTGGT |
|  |  |  | PPD2-R | ACCACGACGCCTACATTGTT |
|  | MET1 | Solyc03g096850.2 | MET1-F | TGTGGCCAGCTGATAAGCTT |
|  |  |  | MET1-R | ACCACAGTCGAGGCAAATGT |
|  |  | Solyc10g047410.1 | 047410-F | ATGCTTGGTCCCGTTCGAAA |
|  |  |  | 047410-R | TCAGCAATACGGCGGTGAAT |
|  | HEMA2 | Solyc01g106390.2 | HEMA2-F | TTCACATGCACTGCTTCGAAAG |
|  |  |  | HEMA2-R | AGCTTCCATTTTCTTCCGCAAC |
|  | GSA1 | Solyc04g009200.2 | GSA1-F | AACGAGTTTCGGTGCTCCAT |
|  |  |  | GSA1-R | TAGGACGGCAAGTGAAAGCA |
|  | HEMC | Solyc07g066470.2 | HEMC-F | TTTGACGACCTTGGATGGGT |
|  |  |  | HEMC--R | AAAGTAGTTCCTGGCCAGCA |
|  | HEME2 | Solyc06g048730.2 | HEME2-F | AGGTCCTTCATTCGCTGCTT |
|  |  |  | HEME2-R | TCTCAAGCAAGCCACCTGAT |
|  | PPOX1 | Solyc01g079090.2 | PPOX1-F | TCGTGATCCGCGTTTACCAA |
|  |  |  | PPOX1-R | TGCTTCGACTTCGCAGAGAA |
|  | CHLH | Solyc04g015750.2 | CHLH-F | AGCGATGCATGTTTCCCAGA |
|  |  |  | CHLH-R | AAGCTGCTTGAGTCCCTTGT |
|  | CHLI | Solyc10g008740.2 | CHlI-F | ATCGCCACTGTCATTCCCAA |
|  |  |  | CHlI-R | AGCTTGGTGCAGGGAAATGT |
|  | CHLD | Solyc04g015490.2 | CHlD-F | TGCCAAAGGAGCAGCACTTA |
|  |  |  | CHlD-R | AACTGCCGTTGTAAGCCCAT |
|  | CHLM | Solyc03g118240.2 | CHLM-F | AAACAGGCGCAAGAGGAGTT |
|  |  |  | CHLM-R | TTCGGTGCAAAGCTCAGGAT |
|  | PORA | Solyc12g013710.1 | PORA-F | TCAAGCTGCTGCATTGCTTC |
|  |  |  | PORA-R | ATGCAACCATTGTCTCGGCT |
|  | DVR | Solyc01g067290.2 | DVR-F | AGGAAGTTTGGGGCTTCACA |
|  |  |  | DVR-R | TGCAAGCACACAACTTTCCA |
|  | CHLG | Solyc09g014760.2 | CHLG-F | CCAATTCCTTCAGGTGCGGT |
|  |  |  | CHLG-R | CCCACCAAGGCAAGCTGATA |
|  |  | Solyc03g115980.1 | Solyc03g115980.1-F | TGGCCAACATACAAGGTGCT |
|  |  |  | Solyc03g115980.1-R | AATGGGGTTTCCTGGTGCAA |
|  | PGI1 | Solyc04g076090.2 | PGI1-F | AGATTCCATGTTCACGCGGT |
|  |  |  | PGI1-R | TCTGCAAACGAGGCTCCATT |
|  | PGM1 | Solyc03g006870.2 | PGM1-F | TGCCACTGGAAGATTTCGGA |
|  |  |  | PGM1-R | TAGCATCTTGGGCATTGGCA |
|  | APS1 | Solyc07g056140.2 | APS1-F | TTGATGGGGGCAGATTACTACG |
|  |  |  | APS1-R | GCCGCTTCTTGAACATTGTCTT |
|  | APL1 | Solyc01g079790.2 | APL1-F | TGCTAAGCCTGCTGTTCCAA |
|  |  |  | APL2-R | TGTGACCCCATTGCCAAAGT |
|  | APL2 | Solyc07g019440.2 | APL2-F | AGGAACAACCAACGGCAGAT |
|  |  |  | APL2-R | ATTATACGTGCGGGCAAGGT |
|  | APL3 | Solyc01g109790.2 | APL3-F | AAGGATGTGGCTGCAGTCAT |
|  |  |  | APL3-R | ACGATTCAGGGCAGCAGAAT |
|  | GBSS | Solyc08g083320.2 | GBSS-F | TGCGATGTTGTTGACCCAGA |
|  |  |  | GBSS-R | TGGCAAGTGGAGCGATTTCT |
|  | SS1 | Solyc03g083090.2 | SS1-F | TCCTGGGAAAATGCAGCCAT |
|  |  |  | SS1-R | ATTCCGCGTCCAATGACAGT |
|  | SS2 | Solyc02g088000.2 | SS2-F | AAGGTGGTTGGGGATTGCAT |
|  |  |  | SS2-R | ACATTGAGGCTTGCCAGTCT |
|  | SS3 | Solyc02g080570.2 | SS3-F | AGAAAGGCTTGCCGAGGAAA |
|  |  |  | SS3-R | TGGGAACATGGATCGTGCAA |
|  | SS4 | Solyc02g071040.2 | SS4-F | TTCCCACGCAAAAATGGTGC |
|  |  |  | SS4-R | ATCAACATCTGGTGGGCGTT |
|  | BE1 | Solyc07g064830.2 | BE1-F | TTCGCATGAAACAGCCTCCT |
|  |  |  | BE1-R | AATTGGCCCATGCAGGAACT |
|  | BE2 | Solyc04g082400.2 | BE2-F | TCGGTGACAAGACCATTGCA |
|  |  |  | BE2-R | TCAGGATGGCCAAACTCGTT |
|  | BE3 | Solyc09g009190.2 | BE3-F | CCACGGCCAAAGAAACCAAA |
|  |  |  | BE3-R | TGTTCCAAAACGGCTGCTTG |
|  | ISA1 | Solyc07g014590.2 | ISA1-F | TGCCCTGTCACGAGTTCAAT |
|  |  |  | ISA1-R | TATTGCACCGAGGCCACAAT |
|  | ISA2 | Solyc09g064800.1 | ISA2-F | ACTTTGAAGGCCGATGCTGA |
|  |  |  | ISA2-R | AAAAGGGAGGGCTGTGTCAA |
|  | GWD | Solyc05g005020.2 | GWD1-F | GCAATTTTGTGGGGATGCCA |
|  |  |  | GWD1-R | TCCATCAAGTCAGCCGCAAT |
|  | PWD | Solyc09g098040.2 | PWD-F | ACATGCTGTAGCCCGTGTTT |
|  |  |  | PWD-R | TGAAGCGAGTGTTTCACCGA |
|  | SEX4 | Solyc03g111160.2 | SEX4-F | TCCTGCAGTTGCGTTGACAT |
|  |  |  | SEX4-R | TCAACGTGACAGGCGTCTTT |
|  | SEX4-2 | Solyc11g007830.1 | SEX4-2-F | TGCGTGCCGAAATAAGGGAT |
|  |  |  | SEX4-2-R | GTTTCGGAAAACAGGAGCGT |
|  | LSF2 | Solyc06g050230.2 | LSF2-F | ATCACTGGAGTGGGCGATTT |
|  |  |  | LSF2-R | TGACCTTTTGTTGGGACCACA |
|  | LSF1 | Solyc12g062250.1 | LSF1-F | AATGGGCGCATGGGAATTTC |
|  |  |  | LSF1-R | TTTTCAGCCTCGATGCCACT |
|  | BAM | Solyc08g077530.2 | BAM-F | TTTGCACAAGCCAAGCCATC |
|  |  |  | BAM-R | CAACGCCATCAAACTCGCAT |
|  | BAM1 | Solyc09g091030.2 | BAM1-F | ATCCTTCAAGCGTCCTCGTT |
|  |  |  | BAM1-R | TGTTCGCGGCATTTGTTAGC |
|  | BAM2 | Solyc08g005780.2 | BAM2-F | TCCAGAAACTGATGACGCGT |
|  |  |  | BAM2-R | TTTGCAGTCTCAACCAGCCT |
|  | BAM3 | Solyc08g007130.2 | BAM3-F | CACACAGCGCGAATTGTTCA |
|  |  |  | BAM3-R | AGTCTGCTCTGCTTGTGCTT |
|  | BAM5 | Solyc07g052690.2.1 | BAM5-F | TGGTACAAAGATGCCAGCCA |
|  |  |  | BAM5-R | AAGCGCATTCTCACCTGCTA |
|  | BAM7 | Solyc01g094580.2 | BAM7-F | TCTGCTGAAAAACCTGCGGA |
|  |  |  | BAM7-R | TTTGCAGCAATGCATGTCCC |
|  | BAM9 | Solyc01g067660.2 | BAM9-F | AGAAGAACGTGTTGCCAGCA |
|  |  |  | BAM9-R | TGACTGCAACTTTCAGCTGC |
|  | AMY1 | Solyc03g095710.2 | AMY1-F | TGCTAACCTTTTGCTCCCAAAA |
|  |  |  | AMY1-R | AACAGAGTGAGATGATGGTGGT |
|  | AMY2 | Solyc04g082090.2 | AMY2-F | TCAATTGGATTGATGGCGCG |
|  |  |  | AMY2-R | TGATGTGAAGGGAAAGGCCA |
|  | AMY3 | Solyc05g007070.2 | AMY3-F | TGCGAAAAACTGGCGTCTTG |
|  |  |  | AMY3-R | TGTTTCCGCTGGATATGGCA |
|  | ISA3 | Solyc06g009220.2 | ISA3-F | ATAAAGGGCGATGCTGGCAT |
|  |  |  | ISA3-R | TTCATTTGCCGTGAACGCAG |
|  | LDA | Solyc11g008050.1 | LDA-F | TTTCGGAAAATGGTGCAGGC |
|  |  |  | LDA-R | ATGAAATGCTCACTGGCGGT |
|  | PHS1 | Solyc03g065340.2 | PHS1-F | TCGGACACCCAAACGTTCTT |
|  |  |  | PHS1-R | TCACGAACACTTTGAGCGGT |
|  | PHS2 | Solyc09g031970.2 | PHS2-F | GGCGAGCTTTGACAAATGCA |
|  |  |  | PHS2-R | TTGGCCAGCCTTCGTGATAA |
|  | DPE1 | Solyc04g053120.2 | DPE1-F | TCTTGCTGGTTGCTCCCTTT |
|  |  |  | DPE1-R | AGAAGCCTCTTTGCTGCCTT |
|  | DPE2 | Solyc02g020980.2 | DPE2-F | ATGGGGCCGGTTTTCTGAAT |
|  |  |  | DPE2-R | ATCCCCAATTCTGGCCGTTT |
|  | MEX1 | Solyc04g064720.2 | MEX1-F | TCTAGCAGGAAATCAAGCTGCA |
|  |  |  | MEX1-R | TTACGGGATTGAGCAAGTGGAA |
|  | GLT1 | Solyc02g086160.2 | GLT1-F | TACGGTGTACATGGGATTTGCA |
|  |  |  | GLT1-R | TGATTCATGAAGCCGGTGTGTA |
|  | GPT1 | Solyc07g064270.2 | GPT1-F | GTTGTGGTCTTTCTGCGCTT |
|  |  |  | GPT1-R | TCCCTCAACAGCAATGGCAA |
|  | GTP2 | Solyc05g045670.2 | GPT2-F | CATGGTGGGCTTTGAATGTTGT |
|  |  |  | GPT2-R | TTTGACATGCTCACTGTTGCAG |
|  | PPT2 | Solyc02g086650.2 | PPT2-F | TACGGCCTCACAATTTGGCT |
|  |  |  | PPT2-R | CAAGAAACAGCGCGGAAAGA |
|  | TPT | Solyc10g008980.2 | TPT-F | AATGGAGTCTCGCGTGTTGA |
|  |  |  | TPT-R | TCCGACTGGTTTTGCCGTAA |
|  | SlDML1 | Solyc09g009080.2 | SlDML1-F | CACAGCAGAAACGAAGGAAGAAG |
|  |  |  | SlDML1-R | GGAGGGTCCTCAATCTTGTTTCT |
|  | SlDML2 | Solyc10g083630.1 | SlDML2-F | GGACTCTGTGGTTGGGGTATATC |
|  |  |  | SlDML2-R | GTTGCACACTCTTCAGGTTCTTC |
|  | SlDML3 | Solyc11g007580.1 | SlDML3-F | GAGAGACACAGCATACACAGACA |
|  |  |  | SlDML3-R | CTTGACGGGAACTTTGTTGAGTC |
|  | SlDML4 | Solyc03g123440.2 | SlDML4-F | TGACTTTGTCTCCACTAGTCACG |
|  |  |  | SlDML4-R | GGGGCACTCTTCAATATCATTGC |
