# Supplementary material for: Potential Role of Domains Rearranged Methyltransferase7 in Starch and Chlorophyll Metabolism to Regulate Leaf Senescence in Tomato
Source: Front Plant Sci. 2022 Feb 8;13:836015. doi: 10.3389/fpls.2022.836015 (PMC8860812; doi:10.3389/fpls.2022.836015)
Supplement: Supplementary file 3 [file Table_2.DOCX]

**Supplementary Table S2. Data description of BS-Seq reads for WT, drm7i_ns-1 and drm7i-1.**

| Sample name | Raw Reads | Raw Bases(G) | Clean Reads | Clean Bases(G) | BS Conversion Rate | Total reads | Mapped reads | Mapping rate | sites_num | sites_covgMean |
| --- | --- | --- | --- | --- | --- | --- | --- | --- | --- | --- |
| WT_1 | 140663020 | 42.2 | 137404348 | 37.66 | 99.40% | 137404348 | 108082260 | 78.66% | 739752703 | 25.75 |
| WT_2 | 111027967 | 33.31 | 108788154 | 29.91 | 99.35% | 108788154 | 87084917 | 80.05% | 739524066 | 21.86 |
| drm7i_ns-1_1 | 126442836 | 37.93 | 123851507 | 34.04 | 99.40% | 123851507 | 97409210 | 78.65% | 739626805 | 24.44 |
| drm7i_ns-1_2 | 136258044 | 40.88 | 133692440 | 36.76 | 99.41% | 133692440 | 105042150 | 78.57% | 739738745 | 25.54 |
| drm7i-1_1 | 114323026 | 34.3 | 112380841 | 30.93 | 99.37% | 112380841 | 90736291 | 80.74% | 739443641 | 22.65 |
| drm7i-1_2 | 133303761 | 39.99 | 131053401 | 36.12 | 99.26% | 131053401 | 108119055 | 82.50% | 739812326 | 28.14 |

WT_1/2 represents WT two replicates; drm7i_ns_1/2 represents drm7i_ns-1 two replicates; drm7i-1 _1/2 represents drm7i -1 two replicates.
