# Supplementary material for: Potential Role of Domains Rearranged Methyltransferase7 in Starch and Chlorophyll Metabolism to Regulate Leaf Senescence in Tomato
Source: Front Plant Sci. 2022 Feb 8;13:836015. doi: 10.3389/fpls.2022.836015 (PMC8860812; doi:10.3389/fpls.2022.836015)
Supplement: Supplementary file 4 [file Table_3.DOCX]

**Supplementary Table 3. Percentage of methylation levels of WT, drm7i_ns and drm7i.**

| Sample | C | mC | mC % | CG | mCG | mCG % | CHG | mCHG | mCHG % | CHH | mCHH | mCHH % |
| --- | --- | --- | --- | --- | --- | --- | --- | --- | --- | --- | --- | --- |
| WT_1 | 254609133 | 90684742 | 35.61% | 24550220 | 20868671 | 85% | 26814731 | 17847529 | 66.55% | 203244182 | 51968542 | 25.56% |
| WT_2 | 254609133 | 84258887 | 33.09% | 24550220 | 20350719 | 82.89% | 26814731 | 17304798 | 64.53% | 203244182 | 46603370 | 22.92% |
| drm7i_ns-1_1 | 254609133 | 82965396 | 32.58% | 24550220 | 20727356 | 84.42% | 26814731 | 17445496 | 65.05% | 203244182 | 44792544 | 22.03% |
| drm7i_ns-1_2 | 254609133 | 85928596 | 33.74% | 24550220 | 20804805 | 84.74% | 26814731 | 17749140 | 66.19% | 203244182 | 47374651 | 23.30% |
| drm7i-1_1 | 254609133 | 87226034 | 34.25% | 24550220 | 20412631 | 83.14% | 26814731 | 17055113 | 63.60% | 203244182 | 49758290 | 24.48% |
| drm7i-1_2 | 254609133 | 90882497 | 35.69% | 24550220 | 21131501 | 86.07% | 26814731 | 17924207 | 66.84% | 203244182 | 51826789 | 25.49% |

WT_1/2 represents WT two replicates; drm7i_ns_1/2 represents drm7i_ns-1 two replicates; drm7i-1 _1/2 represents drm7i-1 two replicates.
